# Antibacterial Properties of Copper Oxide Nanoparticles (Review)

**DOI:** 10.3390/ijms252111563

**Published:** 2024-10-28

**Authors:** Sergey V. Gudkov, Dmitry E. Burmistrov, Polina A. Fomina, Shamil Z. Validov, Valery A. Kozlov

**Affiliations:** 1 Prokhorov General Physics Institute of the Russian Academy of Sciences, Vavilove St. 38, 119991 Moscow, Russia; dmitriiburmistroff@gmail.com (D.E.B.); polja.fomina@gmail.com (P.A.F.); v.kozlov@hotmail.com (V.A.K.); 2 Institute of Biology and Biomedicine, Lobachevsky State University of Nizhny Novgorod Institute, Gagarin Av. 23, 603105 Nizhny Novgorod, Russia; 3 Federal Research Center Kazan Scientific Center of the Russian Academy of Sciences, ul. Lobachevskogo 2/31, Tatarstan, 420088 Kazan, Russia; sh.validov@knc.ru

**Keywords:** CuO, copper oxide, copper oxide nanoparticles, antimicrobial agent, green synthesis

## Abstract

The use of metal and metal oxide nanoparticles is frequently regarded as a potential solution to the issue of bacterial antibiotic resistance. Among the proposed range of nanoparticles with antibacterial properties, copper oxide nanoparticles are of particular interest. Although the antibacterial properties of copper have been known for a considerable period of time, studies on the effects of copper oxide nanomaterials with respect to biological systems have attracted considerable attention in recent years. This review presents a summary of the antibacterial properties of copper oxide nanoparticles, the mechanisms by which the antibacterial effect is realized, and the key reported methods of modifying these nanoparticles to improve their antibacterial activity. A comparative analysis of the effectiveness of these nanoparticles is presented depending on the type of microorganism, the shape of the nanoparticles, and the Gram classification of bacteria based on data from published sources. In addition, the review addresses the biological activities of copper oxide nanoparticles, including their antifungal and cytotoxic properties, as well as their “antioxidant” activity. According to the conducted analysis of the literature data, it can be concluded that copper oxide nanoparticles have a significant bacteriostatic potential with respect to a wide range of microorganisms and, in some cases, contribute to the inhibition of fungal growth. At the same time, the sensitivity of Gram-positive bacteria to the effect of copper oxide nanoparticles was often higher than that of Gram-negative bacteria.

## 1. Introduction

The emergence of antibiotic resistance among bacterial pathogens represents a significant global health concern in the contemporary era. The increasing prevalence of antibiotic-resistant bacterial strains has led to a rise in the incidence of bacterial infections and associated morbidity and mortality globally. This has prompted the investigation of novel alternative antibacterial agents, including those based on nanoparticles (NPs) [1]. Nanoparticles possess a number of distinctive physical and chemical properties, largely attributable to their elevated surface-to-volume ratio. Metal and metal oxide-based nanoparticles have been the subject of considerable research interest over the past few decades due to their accessibility, potential for surface modification, and high reactivity [2,3,4]. In our previous review articles, we provided a comprehensive analysis of the antibacterial properties of a range of metal oxide nanoparticles: zinc oxide NPs [5], iron oxide NPs [6], aluminum oxide NPs [7], and silver oxide NPs [8]. Copper oxide nanoparticles (CopOx-NPs) represent another promising candidate with respect to their antibacterial properties.

The antibacterial properties of copper have been known to humans since ancient times. For example, copper sulfate was added to water for its sterilization and treatment of infections as early as 2400 BC [9,10]. Copper is a metal of low abundance on Earth, ranking 26th in abundance [11]. It is a chemically inactive metal. In dry air and at room temperature, it is weakly oxidized; however, at high humidity and in the presence of CO_2_, it forms the compound hydroxy copper(II) carbonate (CuOH)_2_CO_3_. Copper is a transition metal that exists in two distinct oxidation states: Cu^2+^ (oxidized) and Cu^+^ (reduced). Of particular interest is the biological role of copper. It is an important chemical trace element, serving as a constituent of enzymes associated with a number of crucial processes, including hydroxylation, oxygen and electron transfer, and oxidative catalysis. Copper is a structural or catalytic cofactor of numerous significant enzymes, including ascorbate oxidase, tyrosinase, cytochrome c oxidase, superoxide dismutase, dopamine β-monooxygenase, and others [12]. Copper is a component of hemocyanin, which is a hemoglobin analog molecule found in mollusks and crustaceans [13]. Another crucial biological function of copper is its involvement in iron deposition in the liver and subsequent hemoglobin synthesis. Conversely, high concentrations of soluble copper compounds have been observed to have toxic effects on living organisms. It is established that chronic intoxication of the human body with copper and its salts during manufacture can result in functional disorders of the nervous system, liver, and kidneys. Impairment of copper metabolism in the human body is associated with pathological manifestations and a number of diseases, including Konovalov–Wilson disease and Menkes disease [14]. Consequently, investigations into the impact of copper-containing materials, including nanoparticles, on biological objects are of significant applied interest.

**Figure 1:**
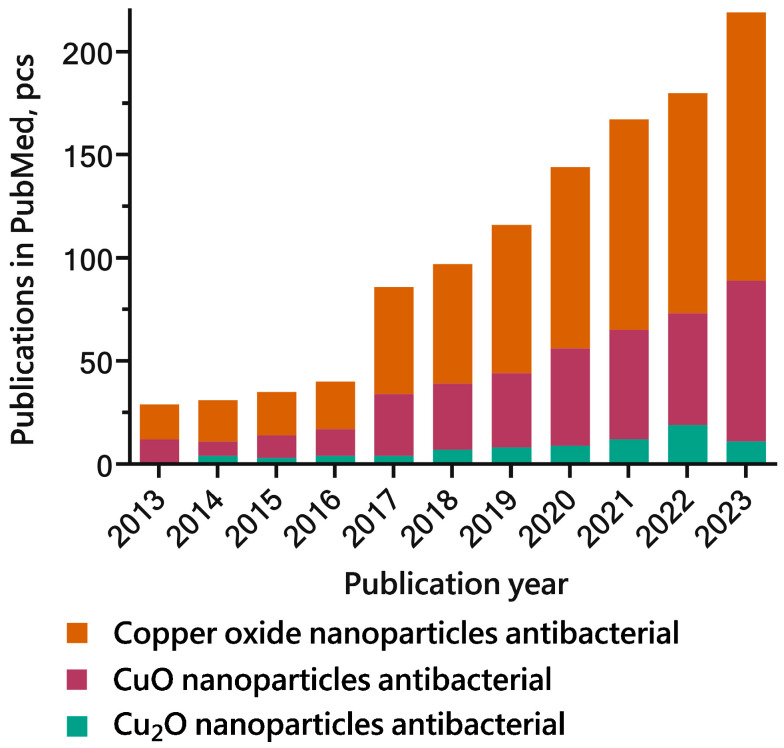
Number of articles published in PubMed investigating the antibacterial properties of copper oxide NPs from 2013 to 2023; the legend displays relevant search terms.

Among nanoparticles of oxides of other metals, CopOx-NPs have recently garnered significant attention due to their distinctive biological, optical, electrical, diamagnetic, and photocatalytic properties [15]. In particular, CopOx-NPs are proposed for use in a number of engineering applications, including as heat carriers in cooling systems [16,17], as semiconductors [18], as gas sensors [19], and as light energy conversion tools [20]. They are also proposed for use in biomedical applications, including as antibacterial [11,21] and biocidal agents [22], in anticancer therapy [23], and as components for intrauterine contraceptives [24]. A review of the published literature reveals a nearly eightfold increase in the number of articles examining the antibacterial properties of CopOx-NPs over the past decade. It is noteworthy that the number of publications devoted to the antibacterial properties of CuO nanoparticles is approximately five and a half times greater than the number of articles describing the efficacy of Cu_2_O nanoparticles (Figure 1). While antibacterial activity has also been documented for Cu_2_O-NPs, both in their pure form [25] and as part of multicomponent materials [26], this review will primarily focus on the antibacterial activity of CuO-NPs due to the limited number of publications investigating the antibacterial properties of Cu_2_O-NPs.

This review presents a synthesis of the findings from the literature on the antibacterial properties of CopOx-NPs. We will examine the principal methods of obtaining these nanoparticles, the mechanisms by which they exert their antibacterial action, the methods of modification that can be used to enhance their activity, and several recent reports on the cytotoxicity of CopOx-NPs with respect to normal human cells, in addition to cell lines of cancer cells. Also, the reported photocatalytic and antioxidant properties of CopOx-NPs, conditioning their possible applications, will be reviewed. The aim of this work was to synthesize the results from the literature on the antibacterial properties of CopOx-NPs over the last 6 years. In separate chapters, we review, in detail, the main methods for obtaining these nanoparticles, the mechanisms by which they exert their antibacterial activity, the modification methods that can be used to enhance their activity, and several recent reports on the cytotoxicity of CopOx-NPs against normal human cells as well as cancer cell lines. The reported photocatalytic and antioxidant properties of CopOx NPs, which determine their potential practical applications, are considered separately.

## 2. Literature Review

### 2.1. Literature Search Process

A systematic search of the academic literature was conducted via the following scientific search engines: Google Scholar, Scopus, and PubMed. The following search terms were used: “copper oxide nanoparticles antibacterial,” “CuO nanoparticles antibacterial,” and “copper oxide nanoparticles antimicrobial.” Additionally, the search was expanded to include the tags “Cu_2_O nanoparticles antibacterial” and “Cu_4_O_3_ nanoparticles antibacterial.” In conducting the search in Google Scholar, the year of publication was restricted to a date later than 2018. As a result of the established criteria, the majority of the articles were published between 2019 and 2024. Publications with a high number of citations were given preference; for example, publications from 2019 to 2021 with at least 30 citations were selected. Furthermore, papers from other review articles on the topic were considered, and the reference lists of other articles, including review articles, containing useful citations and sources were analyzed. In total, the research team conducted a comprehensive review of over 100 publications, the experimental data from which were categorized and summarized in the form of a table (Table 1).

**Figure 2:**
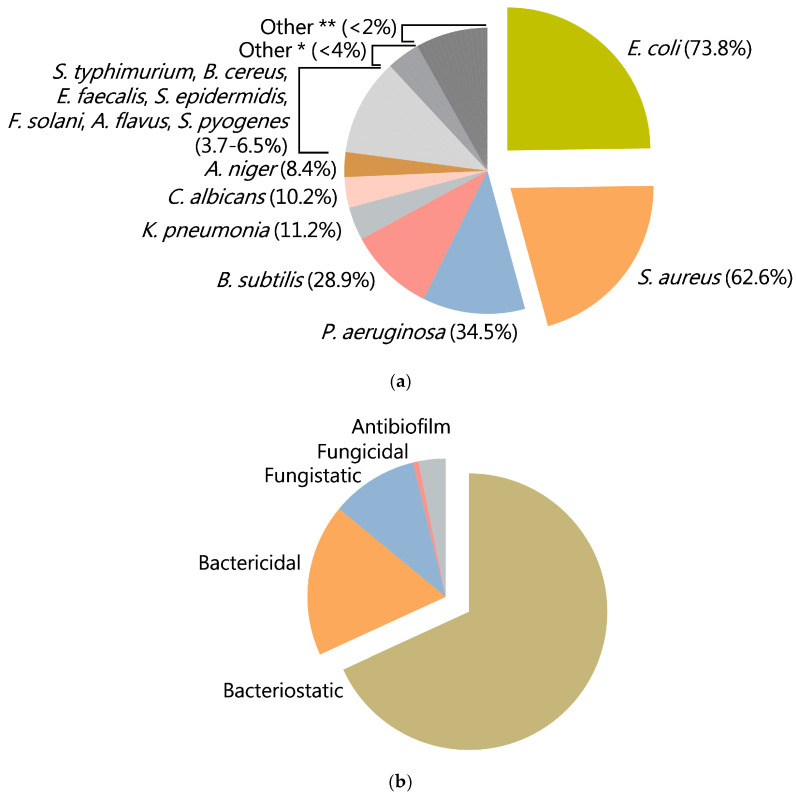
Diagram illustrating the distribution of CuO-NPs-sensitive microorganisms reported in the literature (**a**); diagram illustrating the distribution of biological effects of CuO-NPs reported in the literature (**b**); *—*H. pylori*, *V. cholera*, *S. mutans*, *P. vulgaris*; **—*A. hydrophila*, *C. tropicalis*, *E. tarda*, *E. aerogenes*, *K. aerogenes*, *A. solani*, *L. monocytogenes*, *B. megatarium*, *P. fluorescens*, *P. mirabilis*, *Proteus* sp., *R. solanacearum*, *Penicillium* sp.

Analysis of the literature data presented in Table 1 showed that CuO-NPs exhibit remarkable efficacy against a wide range of bacterial species, including both Gram-positive and Gram-negative strains, as well as several fungal species, including phytopathogenic fungi [37,129,134] (Figure 2a,b).

### 2.2. Methods of Synthesis of CopOx-NPs

Due to the diversity of the reported approaches for obtaining CopOx-NPs, it is possible to control the physical parameters of the synthesized nanomaterials, as well as their morphological characteristics (size, shape). The variability of these parameters allows improving the physicochemical parameters, including the degree of aggregation and the surface properties of NPs, which allows for regulating their biological action, including antibacterial properties.

#### 2.2.1. Traditional Methods

There are numerous techniques for preparing CopOx-NPs, including both physical and chemical methods. In particular, the synthesis of CopOx-NPs has been reported using a variety of methods, including deposition [33], laser ablation [128,135], the sol–gel method [117], mechanochemical processing [54], plasma technology, solvothermal [131], sonochemical [101], and microwave synthesis [56]. The diversity of methodologies employed for the synthesis of CopOx-NPs enables the production of NPs with varying sizes and morphologies. The most commonly synthesized forms of CopOx-NPs are spherical and quasi-spherical. However, other forms have also been reported, including rod-shaped NPs [49,56,74,105], cubic NPs [89,131], rice-like NPs [77], and flake-like NPs. The use of nanoparticles with complex morphology (sponge-like, flake-like, flower-like, and so on) may have a more pronounced antimicrobial effect due to increasing the specific surface area.

**Figure 3:**
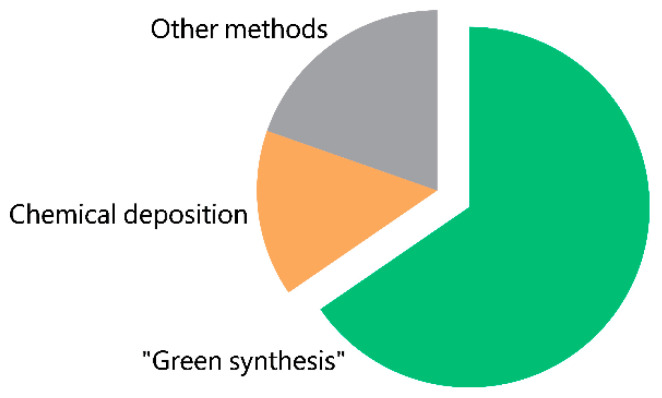
Diagram illustrating the distribution of used methods for the synthesis of CuO-NPs reported in the literature.

The utilization of biological entities, including microorganisms, plants, fungi, and their extracts, for the synthesis of CopOx-NPs represents a significant area of interest for researchers. Among the sources analyzed, approximately 65% of studies employed “green methods” for the synthesis of CopOx-NPs (Figure 3).

#### 2.2.2. “Green Methods”

In addition to using traditional chemical and physical methods to produce colloidal solutions of nanoparticles, “green methods” have recently been increasingly favored to provide a sustainable and cost-effective alternative by minimizing the generation of chemical pollutants during the production of CopOx-NPs [15].

Green chemistry employs a chemical reaction between extracted intracellular components acting as a reducing agent and added copper salts, most often CuSO_4_·5H_2_O, to achieve the desired result. In the case of plant extracts, the alkaloids, phenols, flavonoids, and terpenoids, as well as citrulline, phytosterolinin, β-carotene, tannins, saponins, glycosides, and other compounds, act as reducing agents [77]. In the event that microorganisms are employed, enzymes and proteins are utilized. In the majority of studies, the incorporation of plant extracts has been shown to enhance the stability of the resulting CopOx-NPs [50]. In our review of the literature, we found that approximately 65% of the articles synthesized CopOx-NPs using biological methods. The most prevalent method of “green synthesis” is the utilization of plant extracts. However, a number of works also employed bacterial and fungal cultures, as well as products of their extracellular matrix and extracts [121]. For example, the synthesis of CopOx-NPs has been reported using a variety of microorganisms, including actinomycetes [31], *Stenotrophomonas* sp. BS95 [46], *Lactobacillus casei* [48,136], *Penicillium chrysogenum* [57], *Streptomyces* MHM38 [58], *Penicillium chrysogenum* [79], *Aspergillus terreus* [90], and *Penicillium chrysogenum* [116], as well as *Bacillus cereus* [126].

A number of studies have reported a more pronounced antibacterial effect of CuO-NPs synthesized by the “green pathway” compared to the classical chemical method. For example, Sabeena et al. reported a more pronounced antibacterial effect of CuO-NPs synthesized using *Salacia reticulate* extract against *Escherichia coli*, *Staphylococcus aureus*, *Enterobacter* sp., *Bacillus subtilis*, and *Pseudomonas aeruginosa*, as well as a more pronounced cytotoxic effect against MCF-7 cancer cells, compared to the use of CuO-NPs obtained by chemical precipitation with NaOH [75]. The synthesis of Cu_4_O_3_-NPs using *Razma* seed extract demonstrated inhibitory effects on the growth of both *S. aureus* and *E. coli*. The zone of inhibition (ZOI) for *S. aureus* was 5 mm for 400 μg/mL and 10 mm for 600 μg/mL, while the ZOI for *E. coli* was 10 ± 0.2 mm for 400 μg/mL and 6 mm for 600 μg/mL [132]. The synthesis of CuO-NPs nanorods using *Asparagus racemosus* resulted in a more pronounced bacteriostatic effect than that observed with gentamycin [74]. In addition to exhibiting pronounced bacteriostatic activity, rice-like CuO-NPs synthesized using *Caesalpinia bonducella* seed extract demonstrated the potential for modifying the surface of a graphite electrode for electrochemical detection of riboflavin in a concentration range of 3.13–56.3 nM with a detection limit of 1.04 nM [77]. The synthesis of CuO-NPs using the plant extract of *Silybum marianum* resulted in a notable bactericidal impact against clinically significant pathogenic bacteria, namely *Enterobacter aerogenes* and *Salmonella typhi*. The zone of inhibition (ZOI) values for these bacteria were 18 ± 1.3 and 17 ± 1.2 mm, respectively [81].

## 3. Mechanisms of Antibacterial Activity of CopOx-NPs

**Figure 4:**
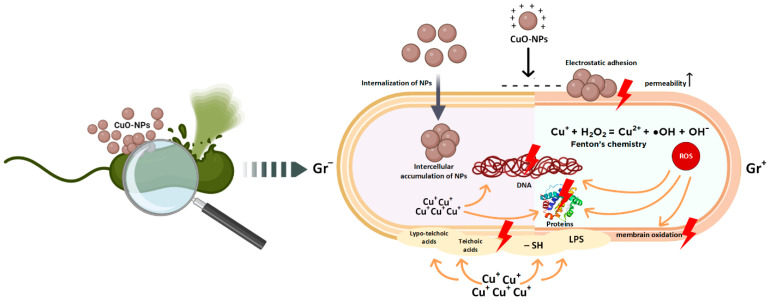
Key mechanisms of antibacterial action of copper oxide nanoparticles according to the literature data.

The primary mechanisms by which CopOx-NPs exert antibacterial activity have been identified as follows: (1) direct interaction between nanoparticles and the surface of bacterial cells, (2) formation of reactive oxygen species (ROS), (3) release of free copper cations into the extracellular and intracellular environments, and (4) interaction of these cations with biomolecules. Additionally, a number of other mechanisms specific to these NPs have been observed (Figure 4). The following section provides a more detailed description of each of these mechanisms.

### 3.1. Contact Interaction of CopOx-NPs with the Surface of Bacteria

The contact interaction of CopOx-NPs with the surface of bacterial cells, as is the case with many other metal and metal oxide NPs, is one of the most frequently reported mechanisms of their antibacterial activity [137]. In the literature, this type of antibacterial effect of nanomaterials is often referred to as “contact killing.” It is well established that the majority of metal nanoparticles and metal oxides possess a positive surface charge. Conversely, the surface of bacterial cells, including both Gram-positive and Gram-negative varieties, is negatively charged. The negative surface charge of Gram-positive bacteria is attributed to the high content of anionic cell wall polymers, including peptidoglycan, which is rich in carboxyl groups of γ-glutamic and meso-diaminopimelic acids, terminal residues of d-Alanine peptide subunits, and teichoic and lipoteichoic acids, which are rich in phosphate groups. In contrast, the negative surface charge of Gram-negative bacteria is attributed to the presence of acidic phospholipids and a limited amount of basic proteins in the outer membrane composition [138]. Due to the action of electrostatic forces, CopOx-NPs are adsorbed onto the surface of bacterial cells, thereby facilitating the destruction of the cell wall. This process increases cell permeability and facilitates the internalization of NPs into the cell [107]. Copper cations (Cu^+^ and Cu^2+^) formed during the leaching of CopOx-NPs can also bind to negatively charged groups of the cell membrane, thereby reducing the membrane potential difference and causing depolarization. This results in damage or rupture of the cell membrane, leakage of cell contents, and, ultimately, death of the bacterial cell [47]. The ultrastructure of bacterial cells exposed to CopOx-NPs has been examined through the use of transmission electron microscopy (TEM) or scanning electron microscopy (SEM) in a number of experimental studies (see Supplementary Materials Figures S1–S3). In particular, Chen et al. demonstrated that CopOx-NPs caused multiple structural damages and aggregation on the surface of soilborne *Ralstonia solanacearum* cells [37]. Halbus et al. [40] demonstrated the extensive accumulation of both pure CopOx-NPs and CopOx-NPs functionalized with GLYMO on the surfaces of *E. coli* cells at a concentration of 25 μg/mL using TEM and SEM. Gopinath et al. demonstrated a change in bacterial cell morphology following a 60 min exposure to CuO-NPs prepared using *Tribulus terrestris* extract [65]. In a recent study by Shehabeldine et al. [79], the ultrastructure of bacterial cells following the application of CuO-NPs was investigated using TEM. An abundant accumulation of CuO-NPs was observed in proximity to the bacterial cells, accompanied by notable alterations in bacterial morphology. These included cell wall ruptures, decreased electron density, a considerable concentration of minute particles within the cell wall and cytoplasm, a loss of smoothness and uniformity in the cell membrane, cell lysis, and the leakage of intracellular material. The ultrastructural analysis of the TEM of bacterial cells incubated with CuO-NPs synthesized using the extract of the fungus *Ganoderma sessile* revealed the accumulation of CuO-NPs on the surfaces of *S. aureus*, *E. coli*, and *P. aeruginosa* [118]. Utilizing scanning SEM, Hesabizadeh et al. observed the attachment of CuO-NPs to the cells of both Gram-positive and Gram-negative bacteria. Additionally, bacterial cells treated with CuO/Cu_2_O-NPs exhibited notable damage to the cell wall and membrane, accompanied by the leakage of intracellular contents and complete cell lysis [107]. Additionally, in the recent work of Rivera-Mendoza et al. [112], it was demonstrated that the growth inhibition of *C. jejuni* upon the addition of CuO-NPs occurred through the attachment of the NPs to the outer cell membrane and subsequent internalization into the cell, a process that was confirmed by TEM.

### 3.2. ROS Release

It is established that transition metal cations, including copper and iron, can facilitate the generation of ROS through Fenton-type and Haber–Weiss reactions. In these reactions, the copper cation participates in the transfer of electrons within the redox cycle between Cu^+^ and Cu^2+^, leading to the formation of highly reactive superoxide anion radicals (O_2_^•−^) and hydroxyl radicals (•OH). These species are capable of causing oxidative damage to proteins, DNA, and lipids [139]. Meghana et al. assessed the impact of the addition of CuO-NPs and Cu_2_O-NPs at concentrations of 0.25 mM and 0.1 mM, respectively, on the generation of AFCs, specifically superoxide anion radical and hydroxyl anions, employing the method of measuring the reduction in NBT to formazan. A notable elevation in superoxide dismutase (SOD) production was discerned in the presence of CuO-NPs, whereas no discernible SOD formation was observed in the presence of Cu_2_O-NPs. In the same study, the impact on hydroxyl radical formation was assessed through deoxyribose reduction estimation. At concentrations of CuO-NPs and Cu_2_O-NPs (0.05 and 0.075 mM), the -OH levels were observed to be higher in the presence of CuO-NPs compared to Cu_2_O-NPs [28]. Additionally, Gopinath et al. employed confocal microscopy with the DCF-DA (2′,7′-dichlorodihydrofluorescein diacetate) dye to demonstrate an intracellular elevation in ROS concentration when cultures of *E. coli*, *P. aeruginosa*, *S. aureus*, and *B. cereus* were incubated with CuO-NPs for one hour [65]. The use of CuO-NPs at a maximum concentration of 19.9 μg/mL against *E. coli* resulted in the formation of ROS at a concentration comparable to that of the positive control (1 mM H_2_O_2_) [118]. Additionally, an increase in intracellular NO concentration was observed following treatment with 0.156–5 mg/mL CuO-NPs. The NO concentration was 1.3 ± 0.03 and 1.6 ± 0.03 µM in the control group and 8 ± 0.9 µM and 8.6 ± 0.45 µM in the HT-29 and AGS cell lines, respectively, following a 72 h treatment with 5 mg/mL CuO-NPs [48]. Also, it is well known that the generation of ROS upon exposure to CopOx-NPs is also a possibility due to photocatalytic reactions, which are discussed in greater detail in the section entitled “Photocatalytic Activity of CopOx-NPs”.

### 3.3. Cation-Mediated Action and Cu-Specific Mechanisms

Another potential mechanism of the antibacterial activity of CopOx-NPs is the release of Cu^+^/Cu^2+^ ions, which subsequently interact with surface molecules of the cell wall and the membrane of the bacterial cell and are absorbed by the bacterial cell. The considerably greater release of Cu^2+^ ions into the medium may be associated with the behavior of the oxide layer on CuO-NPs and the reaction with Cl^−^ ions in the medium [57,140]. Bezza et al. demonstrated the dissolution of Cu^+^ ions from Cu_2_O-NPs, with a significantly higher Cu^+^ concentration observed at pH 5 compared to pH 7 [126]. The excessive accumulation of copper cations in the intracellular space may contribute to Cu^+^/Cu^2+^ competition for metal binding sites in metalloproteins, such as iron [141]. Copper ions released from CopOx-NPs have been observed to actively bind to thiol groups (-SH) of bacterial cell surface proteins, leading to their inactivation and disruption of bacterial membrane permeability, which ultimately results in cell death [54,142]. Due to their high affinity for thiolates, Cu^+^ cathodes have the potential to destabilize Fe-S clusters [10]. Additionally, it has been demonstrated that Cu^2+^ ions, but not Cu^+^ ions, exert direct effects on the F0F1-ATPase of *E. coli*, resulting in conformational alterations and an increase in the number of accessible SH groups [142]. The high sensitivity of bacterial cells to CuO-NPs may be attributed to the presence of an excess of carboxyl groups and amines on their surfaces, which exhibit a high affinity for Cu cations. Additionally, Cu^2+^ has been demonstrated to exhibit a high affinity for double-stranded DNA [143]. Additionally, Cu^2+^ ions that accumulate in the intracellular space have the potential to bind to double-stranded DNA, interfering with its helical structure and resulting in damage. This binding can occur at the phosphate groups, as well as at the large and small grooves within the DNA helix [144].

### 3.4. Photocatalytic Activity of CopOx-NPs

**Figure 5:**
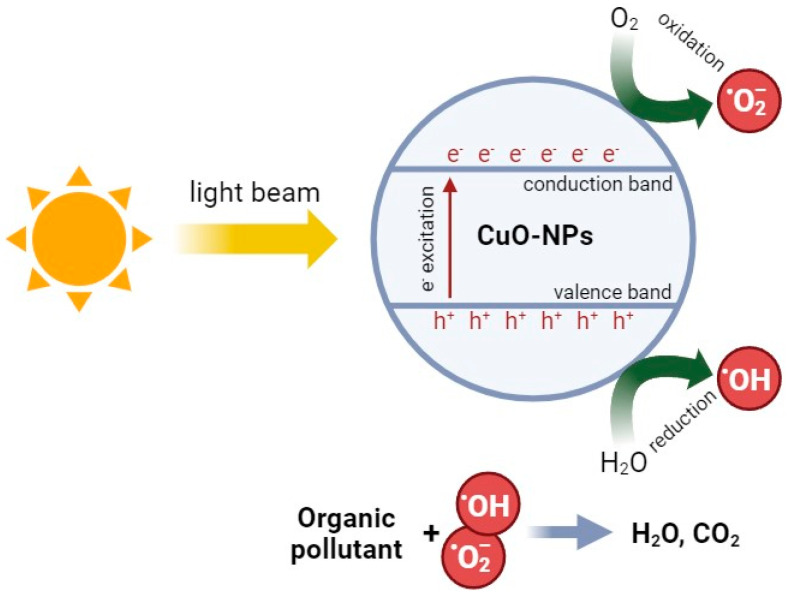
Generalized scheme of photocatalytic reactions for CuO-NPs.

Another biologically useful physical property of CopOx-NPs is their photocatalytic activity [145]. A substantial body of research has proposed the use of these nanoparticles as a solution for the treatment of wastewater containing organic and inorganic pollutants. CuO is a semiconductor with a narrow forbidden energy zone (~1.6 eV), rendering it active in the visible wavelength region of the electromagnetic spectrum [20]. The general mechanism of ROS formation in the course of photocatalytic reactions on the surface of CopOx-NPs can be described as follows: irradiation of CopOx-NPs with light in the visible range leads to the generation of electron–hole pairs in the volume of NPs. Photons of incident radiation promote electrons from the valence band (VB) to the conduction band (CB) of the semiconductor, leaving holes (h^+^) in the VB. The electrons (e^−^) and holes (h^+^) generated on the surface of CopOx-NPs react with water and dissolved oxygen to form ROS. The presence of excited electrons in the CB results in a reduction in oxygen molecules to superoxide anion radicals (O_2_^•–^) and a reduction in H_2_O_2_ to hydroxyl radicals (·OH), which further contribute to the oxidative decomposition of organic molecules or dyes to CO_2_, H_2_O, and other products [103,146] (Figure 5).

In the work of Eid et al., the proposal was made to use CuO-NPs synthesized with the use of an aqueous extract of *Portulaca oleracea* for the decolorization of tannery wastewater under sunlight irradiation. The decolorization efficiency at a CuO-NPs concentration of 2.0 mg/mL, after 200 min of sunlight irradiation, was 88.6 ± 1.5%. The physicochemical parameters of the treated wastewater, including total suspended solids (TSS), total dissolved solids (TDS), chemical oxygen demand (COD), biological oxygen demand (BOD), and conductivity, were significantly reduced by 95.2, 86.7, 91.4, 87.2, and 97.2%, respectively, under optimal conditions [51]. Prakash et al. also put forth the use of CuO-NPs for the remediation of contaminated wastewater, wherein these NPs served as a catalyst in the Biginelli reaction, facilitating a rapid and high yield of 3,4-dihydropyrimidinones. Additionally, CuO-NPs were observed to effectively degrade bromothymol blue (BTB) through photocatalysis, resulting in the formation of hydrogen peroxide. The complete removal of the dye was achieved when BTB was exposed to natural sunlight for a period of three hours [60]. A recent study has also demonstrated the photocatalytic efficiency of CuO-NPs synthesized using *Pterolobium hexapetalum* leaf extract. The successful photocatalytic decomposition of the dye Reactive Black 5 by 98% within two hours of irradiation with UV light (382 nm) was demonstrated, confirming the potential of these NPs for water purification [62].

One of the most prevalent laboratory techniques for evaluating the photocatalytic activity of a substance is the utilization of aqueous solutions of dyes, with methylene blue (MB) being one of the most frequently employed [147]. The degree of MB degradation is assessed by evaluating the optical absorption using a UV-Vis spectrometer at wavelengths of 650–670 nm [148]. In particular, the work of Muthuvel et al. [55] investigated the photocatalytic activity of CuO-NPs synthesized using an aqueous extract of *C. sebestena* flowers. This was achieved by evaluating the degradation of MB upon exposure to sunlight. The degradation of 85 and 97% of the dye was observed after 50 min of incubation with CuO-NPs prepared by classical chemical precipitation and using the plant extract, respectively. In a separate study, CuO-NPs synthesized using *Camellia sinensis* and *Prunus africana* extracts were observed to promote the photocatalytic degradation of MB, with degradation efficiencies of 85.5% and 83.2%, respectively, after 180 min [93]. Another study [69] also demonstrated the photocatalytic action of CuO-NPs synthesized using *Azadirachta indica* extract as a surfactant, with the use of MB under sunlight irradiation. The photocatalytic activity in the presence of CuO-NPs with a concentration of 0.10 g/L was found to be 99%. CuO-NPs synthesized using *Mussaenda frondosa* extract promoted the photocatalytic degradation of MB under UV light with an efficiency of 97.36% within 140 min [71]. The photocatalytic degradation of MB was investigated using copper oxide nanoparticles (CuO-NPs) prepared using the extract of the *Madhuca longifolia* plant. The efficiencies were approximately 77% and 46% for 150 min, using these NPs of sizes 100 and 30 nm, respectively [80]. CuO-NPs synthesized using *Tinospora cordifolia* extract promoted photocatalytic degradation of MB in the presence of both UV and sunlight for 120 min with an efficiency of 91.23% at pH = 2 [83]. CuO-NPs CuO synthesized using *Bergenia ciliata* rhizome extract also showed photocatalytic activity for degradation of MB and methyl red; the efficiency was 92–85% during 135 min of sunlight irradiation [103]. CuO-NPs prepared using *Ocimum tenuiflorum* extract promoted the photocatalytic degradation of methyl orange (MO) by 96.4 ± 0.83% for 24 min at λ = 465 nm [72]. In another work, Sharma et al. [73] also demonstrated photocatalytic cleavage of MO using CuO-NPs synthesized using *Aloe vera* extract. The results were comparable to the previous study. CuO-NPs synthesized using *Psidium guajava* leaf extract demonstrated photocatalytic degradation of Congo red and MB with efficiencies of about 89 and 81% for MB and congo red, respectively, during 160 min of irradiation [44].

The photocatalytic properties of CopOx-NPs make these nanoparticles promising for various applications such as water purification, decomposition of organic pollutants, and other biosafety-oriented technologies. Despite the large amount of available data illustrating the possibility of using CopOx-NPs as photocatalysts, research in this area continues, and new approaches for the synthesis and functionalization of CopOx-NPs can significantly expand the practical applications of these NPs and improve their efficiency.

## 4. Ways to Improve Antibacterial Efficacy of CopOx-NPs

### 4.1. Functionalization of the CopOx-NPs Surface

Various surface modification methods are often considered for metal nanoparticles and metal oxides, such as chemical modification, attaching organic molecules, including biomolecules, forming complexes, or using various physical and chemical treatments. In order to enhance the antibacterial activity, a number of works have proposed to modify the surface of synthesized CopOx-NPs. For example, CuO-NPs functionalized with 4-HPBA (4-hydroxyphenylboronic acid) had the ability to actively accumulate on the surface of bacterial cells, in contrast to non-functionalized CuO-NPs. This effect was attributed to the formation of covalent bonds between the boronic acid groups and the diol groups of the bacterial cell wall glycoproteins. It is noteworthy that the antibacterial activity against *Rhodococcus rhodochrous* and *E. coli* in CuO-NPs functionalized with 4-HPBA was 1 order of magnitude higher compared to pure CuO-NPs [40].

Another effective method for surface modification of CopOx-NPs is functionalization with antibodies. For example, a recent work by Ontiveros-Robles et al. investigated the functionalization of CuO-NPs with monoclonal antibodies specific for Gram-positive and Gram-negative bacteria (*B. subtilis*, *E. coli*). A differential, gram-specific antibacterial effect was observed; the minimal inhibitory concentration (MIC) was 1300 μg/mL for *B. subtilis* and 850 µg/mL for *E. coli* [47].

Another promising method to enhance the antibacterial properties of CopOx NPs is polymer coating. For example, Chen et al. investigated the antibacterial properties of CuO-NPs coated with polyvinyl alcohol (PVA), polyvinyl pyrrolidone (PVP), dopamine hydrochloride (PDA), and polyethylene glycol (PEG) against *E. coli*. CuO-PDA NPs showed the most pronounced bacteriostatic activity compared to other polymer-modified CuO NPs. The authors attributed this effect to the smaller size of these nanoparticles and the lipophilic properties of CuO-PDA-NPs surfaces at pH 7.4, which increased the affinity of these NPs to the lipopolysaccharide-rich outer membrane of *E. coli* [120]. Halder et al. also considered coating CuO-NPs with capsule polymeric substances (CPS) produced by *Bacillus altitudinis* SORB11 as a stabilizing agent. These CuO-NPs at a concentration of 5 μg/mL had significant antibacterial properties against *P. aeruginosa*, and accumulation of CuO-NPs inside *P. aeruginosa* cells was observed. It is noteworthy that the coating of CuO-NPs with CPS is also designed to reduce the negative cytotoxic and genotoxic effects of CuO-NPs through the antioxidant activity of CPS [66]. In turn, the use of lipopeptide surfactants as stabilizers and coating agents for Cu_2_O-NPs was proposed by Bezza et al. these NPs showed bactericidal activity against both Gram-positive *B. subtilis* and Gram-negative *P. aeruginosa* bacteria with MIC of 62.5 μg/mL at pH = 5 and MBC of 125 μg/mL [126].

For Cu_2_O-NPs/biochar nanocomposites, a significant increase in specific surface area was shown compared to pure Cu_2_O-NPs, and the antibacterial properties of Cu_2_O-NPs/biochar nanocomposites were significantly superior to those of Cu_2_O-NPs (MIC was 56 and 40 μg/mL for Cu_2_O-NPs/biochar nanocomposites and Cu_2_O-NPs, respectively) [130]. The functionalization of Cu_2_O-NPs with zeolite A was also proposed. The resulting nanocomplexes inhibited the growth of *E. coli* colonies with an efficiency of 96.19% [127]. Thus, the functionalization of the surface of CopOx-NPs can be aimed not only at increasing the stability and dispersibility of the nanoparticles but also at achieving higher specificity for microorganisms by increasing the specific surface area, the ability to provide high affinity for bacterial cell surfaces, as well as Gram specificity.

### 4.2. Doping with Other NPs/Ions

Another method to enhance the antibacterial properties of CopOx-NPs is doping with other metal ions and doping the NPs with other metals and metal oxides. In particular, CuO-NPs doped with zirconium exhibited bacteriostatic activity against *Enterococcus faecalis* strains ATCC 29212, *Streptococcus mutans* ATCC 35668, *E. coli* ATCC 25922, and a clinical isolate of *Stenotrophomonas maltophilia*. Increasing the Zr content of the complexes contributed to the enhancement of the antimicrobial properties; at 7% Zr in CuO NPs, the ZOI sizes were 12, 18, 10, and 13 for *E. faecalis*, *S. mutans*, *E. coli*, and *Stenotrophomonas maltophilia*, respectively [95]. Also, Thakur et al. [56] demonstrated the bacteriostatic activity of CuO-NPs doped with Ag and Ni against multidrug-resistant bacterial strains, namely *B. subtilis* (MTCC 441), *S. aureus* (MTCC 737), *E. coli* (MTCC 739), and *P. aeruginosa* (MTCC 1688). Compared to pure CuO NPs, Ag and Co ion-doped CuO-NPs were more effective against *S. aureus*, *B. subtilis*, *E. coli*, and *P. aeruginosa* [106]. The bactericidal and bacteriostatic effect of Cu/Cu_2_O NPs synthesized using *Stachys lavandulifolia* extract against *P. aeruginosa* was observed [125]. For nanocomplexes composed of Se and CuO-NPs synthesized using *Punica granatum* peel, a significant bacteriostatic effect was demonstrated against clinical isolates of *Helicobacter pylori* with multiple antibiotic resistance. The effect was more pronounced with Se/CuO NPs compared to Se-NPs [100].

## 5. Comparison of Antibacterial Activity of CopOx-NPs

### 5.1. Spectrum of Microorganisms Sensitive to CuO-NPs Exposure

**Figure 6:**
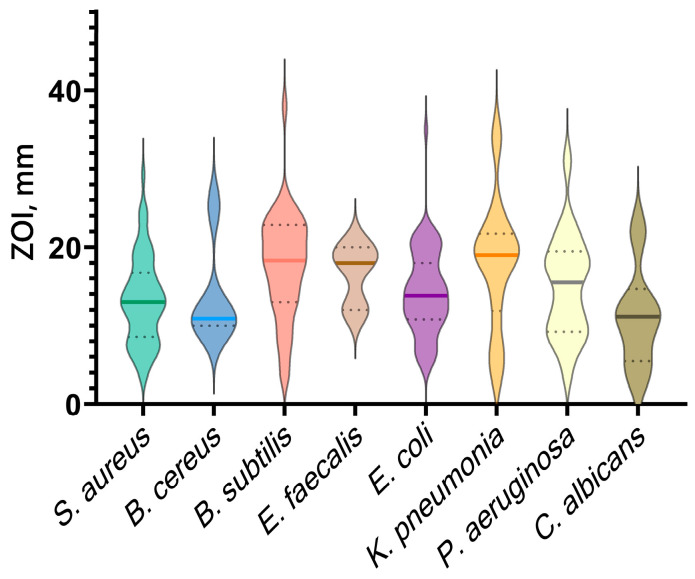
Sensitivity to CuO-NPs of microorganisms, based on the literature data. ZOI—zone of inhibition.

Analysis of the literature data revealed a wide range of Gram-positive and Gram-negative bacterial species, as well as some fungal species, that are sensitive to CuO-NPs (Table 1). The graph (Figure 6) shows the reported ZOI (zone of inhibition) values for the most commonly studied microorganism species. For all microorganism species considered, the average size of the zone of inhibition is in the range of 10–20 mm. The most pronounced sensitivity to CuO-NPs is observed for *Bacillus subtilis*, *Enterococcus faecalis*, *Pseudomonas aeruginosa*, and *Klebsiella pneumonia*. It should be noted that the disk diffusion method often used by the authors of the studies has a number of limitations that make it difficult to compare the results obtained with other works. For example, the results of disk diffusion analysis may vary depending on the composition of the agar medium, its density, and the uniformity of filling the Petri dish. Often, inhomogeneities or errors in the preparation of the medium can affect the accuracy of the measurements. It should also be noted that different bacterial strains may have different sensitivity thresholds, making the interpretation of inhibition zones a complex and standard-dependent task. Therefore, we also compared the antibacterial efficacy of CuO NPs application based on reported minimum inhibitory concentration (MIC) data for Gram-positive and Gram-negative bacterial species; the results of this analysis are presented in the next section.

### 5.2. Gram Specificity

In a number of studies, differences in the antibacterial activity of CuO-NPs were observed depending on the bacterial Gram type. For example, CuO-NPs synthesized using *Thymbra spicata* extract inhibited the growth of *S. aureus* (ZOI 15.25 ± 0.13 μg/mL, MIC 200 µg/mL) and *B. cereus* (ZOI 10.93 ± 0.12 mm, MIC 200 µg/mL) more effectively than *E. coli* and *S. typhimurium*, for which no significant inhibition zones were detected [39]. Also, in the work of Moniri Javadhesari et al., a more pronounced inhibitory activity of CuO NPs against *S. aureus* was observed compared to *E. coli* (MIC 2.50 and 3.75 µg/mL, respectively) [54]. According to some authors, the more pronounced antibacterial effect of CopOx-NPs against Gram-positive bacteria may be due to the difference in cell wall structure between Gram-positive and Gram-negative bacterial species. Copper ions are thought to have a higher affinity for proteins than for lipids, so Gram-positive bacteria with a higher content of peptidoglycan and protein in the cell wall are more susceptible to CopOx-NPs [149].

On the other hand, a number of papers have shown the opposite effect. For example, Shehabeldine et al. [79] reported that *Klebsiella oxytoca* and *E. coli* were more susceptible to CuO-NPs with MIC values of 6.25 and 3.12 μg/mL, respectively, while *S. aureus* and *B. cereus* the MIC value was 12.5 and 25 μg/mL, respectively. The bacteriostatic effect of CuO-NPs doped with rare earth metals (Ce and Nd) was more pronounced compared to pure CuO-NPs and was stronger against Gram-negative *E. aerogenes* compared to Gram-positive *B. subtilis* bacteria [105]. The application of CuO-NPs at a concentration of 45 μg/mL had the most pronounced bacteriostatic effect against Gram-negative species compared to Gram-positive species. Efficacy against the bacterial cell species tested was as follows: *P. aeruginosa* > *E. coli* > *B. subtilus* > *S. aureus* [92]. The bacteriostatic effect of sonochemically synthesized CuO NPs at a concentration of 25 μg/mL was more pronounced against *S. typhimurium* than against *S. aureus* [101]. Similarly, in another work, using CuO-NPs synthesized using the leaf extract of *Averrhoa carambola*, a more pronounced bacteriostatic effect was found against Gram-negative bacterial species *E. coli* and *P. aeruginosa*. A concentration of 100 μg/mL of CuO-NPs was bactericidal against all species tested [122]. Many researchers attribute this effect to the fact that Gram-positive bacteria have a thicker peptidoglycan layer (20–80 nm) compared to Gram-negative bacteria (5–10 nm), which prevents metal ions and NPs from internalizing into the cell membrane and intracellular space [105,122,150].

### 5.3. Effect of Shape and Size of CopOx-NPs on Antibacterial Properties

**Figure 7:**
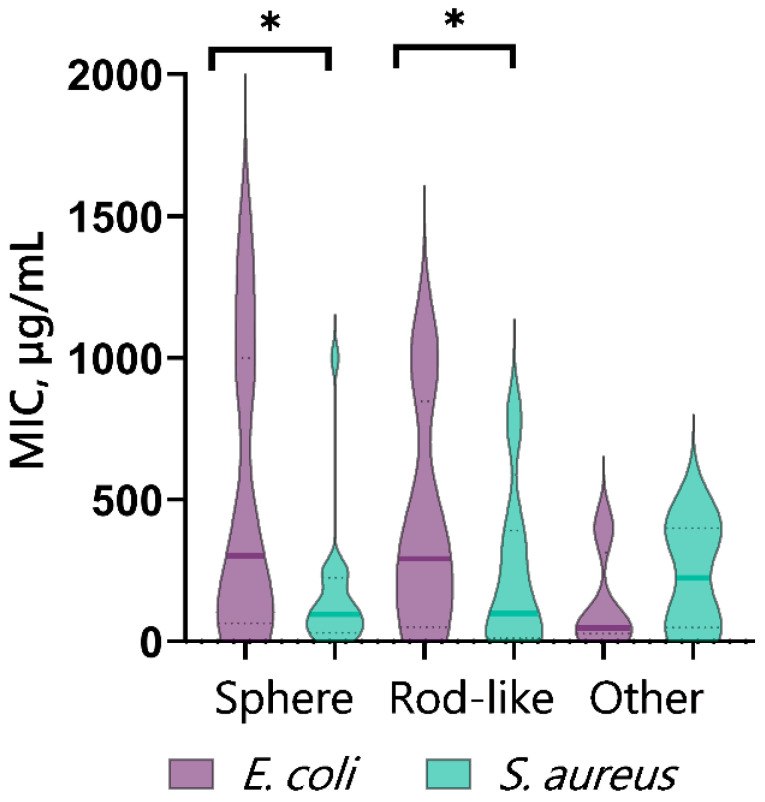
Dependence of bacteriostatic activity of CuO-NPs (MIC) on nanoparticle shape, according to published data, * indicate a significant difference at 5% level in comparison with the control (*p* < 0.05).

During the analysis of the literature data, we compared the reported bacteriostatic activity of CuO-NPs against Gram-positive (*S. aureus* as an example) and Gram-negative (*E. coli* as an example) bacteria when exposed to different forms of these nanoparticles—spherical, rod-shaped (as the most common), and “other”. (Figure 7). There was a decrease in the average MIC value for *S. aureus* both when spherical NPs were added and when rod-shaped CuO NPs were used. The results obtained by analyzing the publications indicate that the sensitivity of Gram-positive bacteria *S. aureus* to CuO-NPs was higher compared to *E. coli*. It is also worth noting the opposite effect for CuO-NPs with “other” morphology, which may be due to the small sample size and insufficient number of reports on the minimum inhibitory concentrations for these nanoparticles.

**Figure 8:**
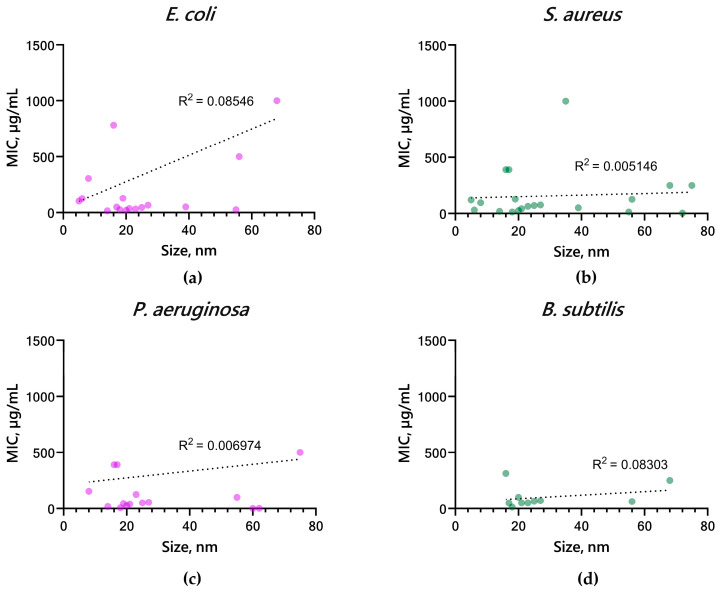
Dependencies of the minimum inhibitory concentration on the size of CopOx-NPs reported in the literature for Gram-negative bacteria (**a**,**c**): *E. coli*, *P. aeruginosa* and Gram-positive bacteria (**b**,**d**): *S. aureus*, *B. subtilis*.

Nanoparticles of smaller size are known to be more reactive due to their larger specific surface area, and consequently, a lower concentration is required to achieve an antibacterial effect. Due to the fact that the activity of nanomaterials may vary with their size, we also analyzed the available data on the bacteriostatic activity of CuO NPs based on published MIC values against Gram-positive (*S. aureus*, *B. subtilis*) and Gram-negative (*E. coli*, *P. aeruginosa*) bacteria as a function of the size of the NPs introduced (Figure 8a–d). For all microorganisms considered, a tendency to decrease MIC (increase in antibacterial potential) with decreasing size of CuO NPs is observed. The graphs also show that the highest density of points is concentrated in the range of 20 ± 10 nm, indicating a tendency to increase the inhibitory activity of CuO-NPs in this size range.

### 5.4. Comparison of Antibacterial Activity of CopOx-NPs with Other NPs

It is interesting to note the comparable antibacterial potential of CopOx-NPs with other NPs in a number of works. In particular, Asamoah et al. compared the efficacy of CuO-NPs and ZnO-NPs against *E. coli* and *S. aureus*. The MIC against *E. coli* and *S. aureus* using CuO-NPs was 1 mg/mL and 0.25 mg/mL, respectively, whereas for ZnO-NPs against *S. aureus*, it was 0.1 mg/mL, and ZnO had no inhibition against *E. coli*. Thus, CuO-NPs possessed higher antibacterial activity as compared to ZnO-NPs [49]. Another study also compared the antibacterial activity of CuO-NPs and ZnO-NPs synthesized using *Mentha pulegium* plant extract against antibiotic-resistant strains of *E. coli* and *S. aureus*. Higher antibacterial activity was also reported for CuO-NPs [82]. Duffy et al. compared the antibacterial activity of three types of nanoparticles (Ag, CuO, and ZnO) against *Salmonella* and *Campylobacter* sp. isolates isolated from poultry at a farm. The efficacy against *Campylobacter* sp. isolates according to the MIC values obtained was as follows: Ag ≥ CuO ≥ ZnO-NPs [85]. Mohamed et al. compared the antibacterial, antibiofilm, and antifungal activities of CuO-NPs and ZnO-NPs synthesized using *P. chrysogenum* filtrate. According to the results obtained, the antibacterial activity against *S. aureus* ATCC23235, *B. subtilis* ATCC6051, *P. aeruginosa* ATCC9027, *S. typhimurium* ATCC14028, *E. coli* ATCC8739 of CuO-NPs was comparable to ZnO-NPs. Both NPs at a concentration of 10 mg/mL inhibited the growth of phytopathogenic fungi (*F. solani*, *F. oxysporum*, *S. sclerotia*, *A. terreus*). However, CuO-NPs were more active in inhibiting the biofilm growth of *S. aureus*. CuO-NPs at concentrations of 0.3, 0.15, 0.07, 0.03, and 0.01 mg/mL reduced biofilm formation by 95, 94.1, 94.4, 85.9, and 68.8%, respectively [116]. It is also interesting to note that the antibacterial effects of CuO-NPs and ZnO-NPs were exhibited depending on the cell growth stage of *E. coli*, *P. aeruginosa*, and *S. aureus* bacterial cultures. In particular, CuO-NPs and ZnO-NPs showed comparable efficacy in the exponential phase of growth, but ZnO-NPs showed almost no activity in the lag phase and had lower efficacy in the stationary phase, while CuO-NPs showed significant bacteriostatic activity in the stationary phase of culture growth [117].

### 5.5. Activity of CopOx-NPs against Biofilm and Resistant Strains

Infections that are associated with biofilm formation are particularly challenging to treat due to their high resistance to physical methods of action and increased resistance to antibiotics. In this regard, the antibiofilm activity of nanoparticles may be of significant interest. It has been demonstrated that CopOx-NPs exhibit activity against both multidrug-resistant bacterial strains and biofilms of pathogens. The synthesis of CuO-NPs using *Centratherum punctatum* leaf extract resulted in the production of nanoparticles with notable antimicrobial activity, which effectively inhibited the formation of biofilms by *P. aeruginosa*. The efficacy was 90.04%, 69.9%, 61.9%, 60.6%, and 71% at concentrations of 4, 2, 1, 0.5, and 0.25 mg/mL, respectively [124]. Additionally, the antibiofilm activity of CuO-NPs at a concentration of 25 μg/mL was demonstrated against *E. coli* and *S. aureus* [41].

A recent study demonstrated that CuO-NPs exhibited a significant inhibitory effect against pathogenic bacterial strains associated with food poisoning and nosocomial infections in humans at concentrations of 25 and 50 μg/mL. *Clostridium perfringens* ATCC 13124, *Clostridium coli* ATCC 33559, *E. coli* ATCC 25922, *Listeria monocytogenes* ATCC 19115, and *Streptococcus pneumoniae* ATCC 13883. The efficacy of CuO-NPs was also demonstrated against other bacterial strains, including *P. aeruginosa* ATCC 27853, *M. catarrhalis* ATCC 25240, *S. aureus* ATCC 25923, *E. coli* ATCC 25922, when compared with the use of tetracycline [59].

Nanocomplexes consisting of selenium (Se) and copper oxide nanoparticles (CuO-NPs) synthesized using *Punica granatum* peel demonstrated a notable bacteriostatic effect against 10 clinical isolates of *H. pylori*, exhibiting resistance to antibiotics, including metronidazole, clarithromycin, levofloxacin, amoxicillin/clavulanic acid, tetracycline, and amoxicillin. The minimum inhibitory concentration (MIC) was 8 μg/mL, which resulted in 100% growth inhibition of resistant *H. pylori*. Moreover, the bacteriostatic effect did not change with increasing concentration of Se/CuO-NPs [100].

The synthesis of CuO-NPs using extracts of *Camellia sinensis* and *Prunus africana* resulted in the production of nanoparticles with bacteriostatic activity against carbapenem-resistant strains of *E. coli* ATCC 96522 and *K. pneumoniae* NTCT 9633, with an MIC of 30 μg/mL [93].

Additionally, CuO-NPs synthesized using the supernatant of *Bacillus siamensis* HS strain have been demonstrated to exhibit antibiofilm activity at higher concentrations of CuO-NPs. At a concentration of 600 μg/mL, CuO-NPs demonstrated the capacity to inhibit the proliferation of biofilms formed by *E. coli*, *P. aeruginosa*, *E. faecalis*, and *Pseudoalteromonas* spp. The most pronounced antibiofilm activity was observed against *Pseudoalteromonas* spp., with an efficacy of 91%. The antibiofilm activity against *P. aeruginosa*, *E. faecalis*, and *E. coli* was 85%, 83%, and 80%, respectively [121].

CuO-NPs, synthesized utilizing the live cell filtrate of the fungus *P. chrysogenum*, demonstrated superior antibiofilm activity against *K. oxytoca* and *E. coli*, reducing biofilm formation of these bacterial species by 49% and 59%, respectively, with MBIC values of 6.25 and 3.12 µg/mL. In contrast, the antibiofilm activity of these CuO-NPs against *B. cereus* and *S. aureus* was comparatively lower, with MBIC values of 200 μg/mL and 256 μg/mL, respectively [79].

High efficacy in the eradication of biofilm-resistant strains of *K. pneumonia* and *H. pylori* was observed with CuO-NPs synthesized using *Cassia fistula* and *Melia azedarach* leaf extracts. The application of CuO-NPs synthesized using *Cassia fistula* extract at a concentration of 1 µg/mL was observed to inhibit the formation of *K. pneumonia* and *H. pylori* biofilms by 99.8% and 100%, respectively. The application of CuO-NPs synthesized using *Melia azedarach* at the same concentration demonstrated a 92.5% and 99.5% suppression of biofilm formation by *K. pneumoniae* and *H. pylori*, respectively. Studies employing SEM demonstrated disruption of bacterial cell morphology and damage to cellular DNA in both species [91].

Consequently, CuO-NPs have been demonstrated to possess notable efficacy in the inhibition of biofilm growth in select bacterial species and have also exhibited activity against antibiotic-resistant strains, including clinical isolates.

## 6. Antifungal Activity of CopOx-NPs

It is known that fungal cells are more resistant to external influences than bacteria due to thick outer and inner cell walls consisting of chitin, glycoproteins, and glucans. As a consequence, the antifungal activity of NPs may be much more difficult to realize than the antibacterial activity [115,151]. Many species of fungi are causative agents of human diseases, also known as mycoses. A separate group of fungi are phytopathogenic species that parasitize crops, reducing the quality of the crop. The possibility of using CopOx-NPs as antifungal agents is often considered along with antibacterial properties [152]. For example, Kamel et al. [129] reported the fungistatic activity of Cu_2_O-NPs against *F. solani*, the causal agent of cucumber root rot. Application of Cu_2_O-NPs, as well as the combined use of Cu_2_O-NPs and fungicide Uniform 390 SE (azoxystrobin + mefenoxam), promoted the activity of catalase, peroxidase and polyphenol oxidase enzymes and the expression of *PR-1* and *LOX-1* genes in cucumber plants (*Cucumis sativus* L.). The antifungal activity of CuO-NPs against *Aspergillus flavus*, *Aspergillus niger*, and *Penicillium oftenans* was shown (ZOI 13.0 ± 1.1, 14.3 ± 0.7, and 16.8 ± 1.4 mm, respectively) [52]. The fungistatic activity of CuO-NPs synthesized using *Polyalthia longifolia* extract against *Aspergillus niger*, *Epidermophyton floccosum*, *Aspergillus clavatus*, and *Candida albicans* was reported (MIC was 1000, 100, 1000, and 500 μg/mL, respectively) [78]. Also, antifungal activity of CuO-NPs against fungal phytopathogens *Fusarium oxysporum*, *Alternaria solani*, *Aspergillus niger* (ZOI 37.0, 28.0, and 26.5 mm, respectively) was reported. Notably, the effect of these CuO-NPs against bacterial plant pathogens *Ralstonia solanacearum* and *Erwinia amylovora* (ZOI 22.0 and 19.0 mm, respectively) was also found [57]. Another work also showed the antifungal activity of CuO-NPs against *Rhizoctonia solani*, *Fusarium solani*, and *Aspergillus niger* [58]. Moreover, the activity against *Candida albicans* was the highest, surpassing even the effect on bacterial cells. The fungistatic effect of CuO-NPs against *Fusarium solani* was observed. The application of these NPs at a concentration of 80 μg/mL prevented ~90% of mycelial growth [63]. A potent fungistatic action was shown against *Candida albicans*, *Candida tropicalis*, *Aspergillus niger*, and *Aspergillus flavus* using CuO-NPs synthesized using *Plectranthus amboinicus* leaf extract [76]. The ability of CuO-NPs synthesized using *Syzygium alternifolium* extract was observed to inhibit the growth of *Alternaria solani* ATCC 32904, *Aspergillus flavus* ATCC 9643, *Aspergillus niger* ATCC 16404, *Penicillium chrysogenum* ATCC 11709, and *Trichoderma harzianum* ATCC 20476 [116]. Another study demonstrated the fungistatic effect of CuO-NPs synthesized using *Azadirachta indica* extract against *Candida albicans* and *Aspergillus niger* [69]. Also, the fungicidal effect of CuO-NPs synthesized using leaf extract of *Aerva javanic*a plant against *C. albicans*, *C. krusei*, and *C. tropicalis* has been reported. The minimum fungicidal concentrations (MFC) of CuO-NPs were 160 μg/mL [70]. Overall, the antifungal activity of CopOx-NPs represents an important area of research due to their potential application in the control of fungal infections.

## 7. “Antioxidant” Properties of CopOx-NPs

Copper is a metal of variable valence, which endows it with notable pro-oxidant properties [153]. It is frequently observed that elevated concentrations of copper within cells give rise to the emergence of oxidative stress [154]. Oxidative stress is associated with damage to nucleic acids, proteins, lipids, and other biomolecules [155,156]. At the same time, there is quite a lot of information in the literature about the “antioxidant” properties of copper nanoparticles. It was demonstrated earlier that CuO-NPs synthesized using *Thymbra spicata* leaf extract actively reduced the optical absorption of 2,2-diphenyl-1-picrylhydrazyl (DPPH) solution [39]. The synthesis of CuO-NPs using *Cissus vitiginea* extract demonstrated a 21% reduction in the optical absorption of DPPH [94]. The synthesis of CuO-NPs using the endophytic fungus *Aspergillus terreus* also demonstrated antioxidant activity with DPPH, with a half maximal inhibitory concentration (IC_50_) value of 40 μg/mL for DPPH [90]. The antioxidant activity of CuO-NPs synthesized using *Plectranthus amboinicus* extract was observed. The DPPH uptake efficiency was 95% using 80 μg/mL CuO-NPs, with an IC_50_ of 40 μg/mL [76]. Manasa et al. observed that CuO-NPs synthesized using *Mussaenda frondosa* extract exhibited strong antioxidant radical scavenging activity, as evidenced by their ability to reduce DPPH [71]. The antioxidant activity of crude and calcined CuO-NPs synthesized using a cell-free extract of *Stenotrophomonas* sp. BS95 was demonstrated by DPPH [46]. The optical absorbance of DPPH when CuO-NPs synthesized using *Solanum nigrum* leaf extract were added to the solution was approximately 10–81% of the control, depending on the concentration. In comparison, chemically synthesized CuO-NPs demonstrated an efficiency of 38–91%, with the range of concentrations of CuO-NPs used being 15–500 μg/mL [55]. CuO-NPs synthesized using *Silybum marianum* extract also showed antioxidant potential in neutralizing DPPH: 55.5 ± 0.62% [81]. Additionally, CuO-NPs synthesized using *Tinospora cordifolia* extract demonstrated efficacy in reducing the optical absorption of DPPH, with an IC_50_ value of 566 μg/mL [83]. The CuO-NPs synthesized using plant extracts of *Camellia sinensis* and *Prunus africana* bark extract demonstrated efficiency in changing DPPH staining by approximately 23% [93]. The synthesis of CuO-NPs using *Bergenia ciliata* rhizome extract also demonstrated an antioxidant effect, with an IC_50_ value of 72.4 μg/mL for DPPH [103]. For Cu_3_O_4_-NPs synthesized using *Razma seed* extract, a significant “antioxidant” activity in DPPH uptake was also found; IC_50_ was 502 μg/mL [132]. What all the above works have in common is the use of DPPH reagent to investigate antioxidant properties. The stable radical DPPH functions as a trap for other radicals, including those of the ROS, but not exclusively so [157]. DPPH in solution exhibits a dark purple coloration with an absorption maximum at 520 nm. Upon neutralization, it assumes a colorless or pale-yellow appearance [158]. It seems plausible that copper oxide nanoparticles or copper ions in solution are capable of interacting with DPPH. It seems prudent to proceed with caution with regard to claims regarding the antioxidant properties of metal oxides.

## 8. Cytotoxicity and Anticancer Properties of CopOx-NPs

The study of the cytotoxicity of nanomaterials is a topic of great interest, as it concerns the safety of the further practical application of synthesized materials. In the case of metal nanoparticles and metal oxides, it is common practice to consider exposure to both cancer cell lines and normal cells. With regard to CopOx-NPs, there is a considerable body of evidence indicating cytotoxicity in vitro, which we discuss in greater detail below.

CuO-NPs synthesized using *Nilgirianthus ciliatus* extract exhibited anticancer activity against human breast cancer cell line (MCF-7) and lung cancer cell line (A549) with minimal cytotoxic effect against fibroblasts. The percentage of viable cells in cultures after exposure to 100 μg/mL CuO NPs after 24 h of in vitro cultivation was 58.97, 59.40, and 81.97% for MCF-7, A549, and L929 cells, respectively, with complete survival of normal cells [61]. CuO-NPs synthesized using *Mussaenda frondosa* extract were also found to be cytotoxic against the A549 cell line after incubation for 48 h (IC_50_ ~1500 μg/mL) [71]. In turn, CuO NPs synthesized using *Salacia reticulate* extract also exhibited cytotoxicity against MCF-7 and less pronounced cytotoxicity against normal HaCat (human keratinocytes) cell lines [75]. The cytotoxic effect of CuO-NPs synthesized using *Thymbra spicata* leaf extract was investigated against L929 cells; in particular, the cytotoxic effect was more pronounced when smaller CuO-NPs were introduced into the culture medium [39]. A significant cytotoxic effect of Se/CuO-NPs was demonstrated against the gastric cancer cell line SNU-16; the IC_50_ was 7.1 ± 0.4 μg/mL, while Se-NPs without CuO-NPs doping showed less pronounced cytotoxic properties (IC_50_ 22.63 ± 1.36 μg/mL) [100]. A significant decrease in cell viability was observed in human gastric cancer cells (AGS) cultures and human colon cancer (HT-29) cell lines when CuO-NPs synthesized using *L. casei* bacteria were administered [48]. The anticancer effect of CuO-NPs against the AGS cell line was also demonstrated in another work, where the IC_50_ was about 50 μg/mL. It was also shown that the death of cancer cells under the influence of these NPs is predominantly through the apoptotic pathway [65]. CuO NPs synthesized from the endophytic fungus *Aspergillus terreus* inhibited the growth of HT-29 cancer cells; IC_50_ was 22 μg/mL [90]. A recent work by Talebian et al. [46] investigated the cytotoxic properties of CopOx-NPs synthesized by the green method using *Stenotrophomonas* sp. BS95 strain against cancer cell lines LoVo (human colon adenocarcinoma), MKN-45 (human gastric adenocarcinoma), and HDF (human dermal fibroblasts). The IC_50_ values using crude CuO-NPs were 48.36, 90.23, and 158.2 for LoVo, MKN-45, and HDF cells, respectively. In contrast, when these NPs were used in dried form, the IC_50_ values were 44.96, 117.5, and 222.8 μg/mL, respectively. It is also interesting to note the reported significant increase in the expression of apoptosis-related genes in these cells upon incubation with CuO-NPs: *P53*, *BAX*, *BCL2*, and *CCND1*. Flores-Rábago et al. investigated the cytotoxic properties of CuO-NPs synthesized using extracellular metabolites and *Ganoderma sessile* mushroom extract against three mammalian cell lines: AML-12 (normal mouse liver hepatocytes), RAW 264.7 (mouse macrophages), and MDCK (dog kidney cells). It was found that at low concentrations of CuO-NPs (<15 μg/mL), cytotoxicity against cells was weakly expressed [118]. The anticancer activity of CuO-NPs synthesized using *Pterolobium hexapetalum* extract against the MDA-MB-231 cell line (breast cancer cells) was also shown; the IC_50_ was 30 mg/mL. At the same time, cytotoxicity against the normal cell line HBL-100 was weaker, and even at the maximum concentration of CuO-NPs 50 μg/mL, the percentage of viable cells was about 84% [62]. Also, the anticancer activity of CuO-NPs synthesized using *Syzygium alternifolium* extract against MDA-MB-231 cell line (IC_50_ 50 μg/mL) was demonstrated in [64]. A dose-dependent cytotoxic effect of CuO-NPs synthesized using *Silybum marianum* extract against NIH3T3 cell line (mouse fibroblasts) was observed; the percentage of cell viability was 83.60 ± 1.5% and 55.1 ± 1.8% at CuO-NPs concentration of 25 μg/mL and 100 μg/mL, respectively, while normal non-immortalized cultures did not lose viability [81]. Cytotoxicity studies of CuO-NPs against the Neuro2A cell line (mouse neuroblastoma) showed moderate cytotoxicity, with an IC_50_ of ~120 μg/mL [70]. Thanuja et al. reported the cytotoxic effects of Cu_4_O_3_-NPs synthesized using *Razma* extract against human prostate cancer cells (PC-3); the IC_50_ was 241.83 μg/mL [132]. Thus, the reported anticancer and cytotoxic properties of CopOx-NPs suggest the possibility of biomedical applications of these nanoparticles. It is also notable that the reported concentrations of CopOx-NPs at which cytotoxicity was observed significantly exceed the concentrations at which antibacterial effects were noted.

## 9. Conclusions

Despite the long-standing recognition of copper’s antibacterial properties, the potential for the safe utilization of copper-based materials has been a subject of contention due to concerns regarding their toxicity. The rapid development of nanotechnology has facilitated the emergence of novel methods for the synthesis and manipulation of nanoscale particles, as well as approaches for their modification and functionalization. The biological activity of copper oxide-based nanoparticles is unquestionable, particularly with regard to their antibacterial properties. The employment of an array of synthesis techniques, particularly those deemed “green” due to the use of plant extracts and components, has enabled the production of copper oxide nanoparticles with the capacity to impede the proliferation of a multitude of microbial species. Additionally, these nanoparticles have demonstrated the ability to prevent the formation of biofilms by resistant strains and clinical isolates. It is also noteworthy that copper oxide nanoparticles, when subjected to various surface modifications and when used in the form of nanocomplexes with other nanoparticles, exhibit high efficiency. The emergence of studies demonstrating the specificity of copper oxide nanoparticles to bacteria depending on their Gram type indicates the necessity for further, more detailed studies aimed at identifying the exact mechanisms of their action and specific molecular targets. Fungal infections are known to exhibit a moderate degree of resistance to chemical and physical methods of action. Consequently, data on the fungistatic action of copper oxide nanoparticles are also of great interest. Moreover, the physical properties of nanosized copper oxide, particularly its photocatalytic activity, offer promising avenues for its practical applications.

Furthermore, it is also noteworthy to mention the documented antiviral efficacy of copper oxide nanoparticles, which has been the subject of numerous recent investigations but was not included in this review. The incorporation of these nanoparticles as functional additives in polymeric materials has the potential to be an efficacious method of creating “self-disinfecting” surfaces. In consideration of the considerable prevalence, frequency of occurrence, and high mortality rate of viral epidemics, this approach may prove to be a highly promising solution.

A substantial body of research has been conducted on the cytotoxic properties of copper oxide nanoparticles, particularly against cancer cell lines. Numerous studies on cytotoxic properties, especially against cancer cell lines, are of great value in view of the potential biomedical applications of copper oxide nanoparticles, especially considering the reported low cytotoxicity against normal cells.

The existing literature data collectively indicate that copper oxide nanoparticles are an attractive and highly promising nanomaterial with a diverse range of biological activities. Nevertheless, further studies are required to assess the effects of these nanoparticles not only at the cellular level but also at the level of individual biomolecules. It is evident that the issue of toxicity at the organismic level remains a significant concern, and it is imperative that further comprehensive investigations be conducted to assess the toxicity of copper oxide nanoparticles and related materials in vivo.

## Figures and Tables

**Table 1:** Antibacterial properties of CopOx-NPs reported in the literature.

No.	Synthesis Method	Composition	Size, nm	Shape	Concentration	Medium, Conditions	Microorganism	Biol Effect	Ref.
1	Electrochemical reduction using tetrabutylammonium bromide	CuO	5–10	Sph.	50–100 µg/mL	NB, 24 h, 37 °C, NA, 1 h	*E. coli*, *S. aureus*	BS	[27]
2	Chemical precipitation followed by drying (for Cu_2_O, the reducing agent hydrazine is added)	Tryptophan-coated CuO, Cu_2_O	30 for CuO, 40 for Cu_2_O	Sph.	0.1 mM, 0.05 mM (MIC); 0.25 mM, 0.1 mM (MBC)	NA, 18 h	*E. coli*	BS, BC	[28]
3	Combustion using ascorbic acid as a coating agent	CuO	36, 45	Sph.	100 µg/mL	NA, 24 h, 37 °C	*E. coli*, *S. typhi*, *M. luteus*, *P. fluorescens*, *S. flexneri*, *V. cholerae*	BS	[29]
4	Colloidal–thermal synthesis (using caraya gum)	CuO	2–10	Sph.	10–100 µg/mL	NB, 18–24 h, 35 °C	*E. coli*, *S. aureus*	BS, BC	[30]
5	Actinomycete-mediated “green synthesis”	CuO	61.7	Sph.	5–100 µg/mL	Agar for the isolation of Actinomycetes and starch-casein agar, 7–14 days, 27 °C	*S. aureus*, *B. cereus*, *P. mirabilis*, *E. tarda*, *A. caviae*, *A. hydrophila*, *V. anguillarum*	BS	[31]
6	“Green synthesis” using *Malva sylvestris* extract	CuO	14	Sph.	–	–	*Shigella*, *Listeria*	BS	[32]
7	Chemical deposition	CuO	23	Sph.	31.25–250 µg/mL (MIC)	NA, 24 h, 37 °C, MHB, 12 h, 37 °C	*E. coli*, *P. aeruginosa*, *K. pneumonia*, *E. faecalis*, *S. flexneri*, *S. typhimurium*, *P. vulgaris*, *S. aureus*	BS	[33]
8	“Green Synthesis” using *Aloe vera* extract	CuO	9–23	Rod, Sph.	10 µg/disk	MHA, 24 h, 37 °C	*E. coli*, *S. aureus*	BS	[34]
9	Thermoplasmic technology	CuO, CuO/Ag	20–95	Sph.	100–5000 µg/mL	TSB, 24 h, 37 °C	*S. aureus*, *S. epidermidis*, *P. aeruginosa*, *E. coli*, *Proteus* sp.	BC	[35]
10	Chemical deposition	CuO	70–90	Rope-shaped	50–200 µg/disk	NA, 24 h, 37 °C	*P. aeruginosa*, *S. aureus*, *B. subtilis*, *E. coli*	BS	[36]
11	“Green synthesis” using *C. papaya* extract	CuO	40–80	Sph.	50–250 µg/mL	NB, 12, 24 and 72 h, 30 °C	*R. solanacearum*	BS, BC	[37]
12	“Green synthesis” using dried goat and sheep fecal matter	CuO	29.20 ± 15.9 (32.30 ± 32.9)	Sph.	100 µg/mL	NA, 24 h	*B. subtilis*, *S. typhimurium*	BS	[38]
13	“Green synthesis” using *Thymbra spicata* leaf extract	CuO-NPs + *Thymbra spicata* extract	26.8, 21	Sph.	0.78–200 µg/mL	MHA, 24 h, 35 °C, MHB, 24 h, 35 °C	*B. cereus*, *S. aureus*, *E. coli*, *S. typhimurium*	BS, AB	[39]
14	Chemical deposition	CuO, CuONPs/GLYMO, CuONPs/GLYMO/4-HPBA (CuO + BA)	13, 106 ± 6 (for CuONPs/GLYMO), 121 ± 4 (for CuONPs/GLYMO/4-HPBA)	–	5–250 µg/mL	LB broth, 10 min., 1 h, 6 h, 37 °C	*S. aureus*, *S. choleraesuis*, *C. albicans*, *P. aeruginosa*, *B. subtilis*	BS	[40]
15	“Green synthesis” using apple peel extract	CuO	25–55	Square	100–300 µg/mL	LB agar, 24 h, 37 °C	*E. coli*, *S. aureus*	BS, BC, AB	[41]
16	Microwave synthesis from starch, glucose, and CuCl_2_	CuO	1.36 ± 0.6	Sph.	0.0113, 0.00113, 0.000113 M/L	NA, 24 h, 37 °C	*E. coli*, *S. epidermidis*, *S. aureus*, *B. megatarium*	BS	[42]
17	“Green synthesis” using *Catha edulis* extract	CuO	28.1, 25.3, 18.2	Sph.	20, 40 mg/mL	MHA, 24 h, 37 °C	*S. aureus*, *S. pyogenes*, *K. pneumonia*, *E. coli*	BS	[43]
18	“Green synthesis” using *Psidium guajava* extract	CuO	40–150	Sph.	20–80 µg/mL	NB, MHA, 24 h, 37 °C	*E. coli*, *P. aeruginosa*, *S. pneumoniae*, *S. epidermidis*	BC	[44]
19	“Green synthesis” using *Catharanthus roseus* extract	CuO	<100	Sph.	10, 30, 50 µL/disk	NA, during night, 37 °C	*S. aureus*	BS	[45]
20	“Green synthesis” using *Stenotrophomonas* sp. strain BS95	CuO	55.92 (crude), 68.35 (calcined)	Sph.	31.25–2000 µg/mL	MHB, 18 h, 37 °C, MHA, 24 h, 37 °C	*B. subtilis*, *S. aureus*, *P. putida*, *E. coli*	BS, BC	[46]
21	Commercially available NPs (Sigma-Aldrich) functionalized with specific antibodies	CuO, CuO-NP-AbGram, CuO-NP-AbGram^+^	242 ± 96 (CuO), 40 ± 42 (CuO-NP-AbGram^+^), 707 ± 243 (CuO-NP-AbGram^-^)	–	MIC: 1250/2250 µg/mL (CuO), 850/2900 µg/mL (CuO-NP-AbGram^-^), 1300/1600 µg/mL (CuO-NP-AbGram^+^)	LB broth, 24 h, 37 °C	*E. coli*, *B. subtilis*	BS	[47]
22	“Green synthesis” using cultures of *Lactobacillus casei*	CuO	40–110	Sph.	0.075–5 mg/mL	MHA, 24 h, 37 °C	*S. aureus*, *P. aeruginosa*	BS, BC	[48]
23	Chemical deposition	CuO	100	Rod.	0.01–5 mg/mL	NHA, MHB, overnight, 37 °C	*E. coli*, *S. aureus*	BS	[49]
24	“Green synthesis” using *Terminalia belerica* extract	CuO	9–14	Sph.	1–10 mg/mL	NA, 24 h, 35 ± 2 °C	*S. aureus*, *B. subtilis*, *E. coli*, *K. pneumoniae*, *S. enterica*	BS	[50]
25	“Green synthesis” using aqueous extract of *Portulaca oleracea*	CuO	5–30	Sph.	3.12–200 µg/mL	MHA, 24 h, 35 ± 2 °C	*S. aureus*, *B. subtilis*, *P. aeruginosa*, *E. coli*, *C. albicans*	BS, FS	[51]
26	“Green synthesis” using *Morinda citrifolia* extract	CuO	20–50	Sph.	15–25 µL/disk	MHA, 24 h, 37 °C (bacteria); Dextrose agar, 24 h, 37 °C (fungi)	*E. coli*, *B. subtilis*, *S. aureus*; *A. flavus*, *A. niger*, *P. frequentans*	BS, FS	[52]
27	Gel combustion method	CuO	20–27	Sph.	10–100 µg/mL	NA, 24 h, 35 ± 2 °C	*E. coli*, *P. aeruginosa*, *B. subtilis*, *S. aureus*	BS, BC	[53]
28	Mechanochemical processing	CuO	7, 14	Sph.	1.25–10 mg/mL	NB, 24–72 h	*E. coli*, *S. aureus*	BS, BC	[54]
29	Chemical sol–gel method/“Green synthesis” using *Solanum nigrum* extract	CuO	32, 25	Sph.	50, 100 µL/disk	NA, 24 h, 35 °C	*B. subtilis*, *S. saprophyticus*, *E. coli*, *P. aeruginosa*	BS	[55]
30	Microwave fusion	CuO, Cu_0.96_Ag_0.02_Ni_0.02_O, Cu_0.94_Ag_0.02_Ni_0.04_O, Cu_0.92_Ag_0.02_Ni_0.06_O, Cu_0.90_Ag_0.02_Ni_0.08_O	16.53–29.81	Rod, Needle, Sph.	10, 25, 50 mg/mL	MHA, 24 h, 37 °C	*B. subtilis*, *S. aureus*, *E. coli*, *P. aeruginosa*	BS, BC	[56]
31	“Green fusion” using *Penicillium chrysogenum*	CuO	9.7–10.7	Sph.	250 µg/mL	NA, 24 h, 37 °C; PDA, 5 days, 28 °C	*E. amylovora*, *R. solanacearum*, *F. oxysporum*, *A. niger*, *P. citrinum*, *E. cichoracearum*, *A. solani*	BS, FS	[57]
32	“Green synthesis” using *Streptomyces strain* MHM38	CuO	1.72–13.49	Sph.	200 mg/mL	MHA, 24 h, 37 °C, potato D-glucose agar/Sabouraud dextrose agar, 120 h, 28 °C	*E. faecalis*, *S. typhimurium*, *P. aeruginosa*, *E. coli*, *R. solani*, *F. solani*, *A. niger*, *C. albicans*	BS, BS	[58]
33	“Green synthesis” using orange, lemon, and tangerine peel extracts	CuO	48–76	Sph.	25, 50 mg/mL	MHA, during night, 37 °C	*E. faecalis*, *S. aureus*, *L. monocytogenes*, *S. pneumoniae*, *C. perfringens*, *E. coli*, *M. catarrhalis*, *S. diarizonae*, *C. coli*, *P. aeruginosa*	BS	[59]
34	“Green synthesis” using *Cordia sebestena* extract	CuO	20–35	Sph.	1000 µg/mL	NA, 24 h, 37 °C	*B. subtilis*, *S. aureus*, *E. coli*, *K. pneumoniae*	BS	[60]
35	“Green synthesis” using an extract of *Nilgirianthus ciliatus*	CuO	20	Sph.	250, 500, 1000 µg/mL	MHA, 24 h, 37 °C	*E. coli*, *P. aeruginosa*, *S. aureus*, *S. mutans*	BS	[61]
36	“Green synthesis” using *Pterolobium hexapetalum* extract	CuO	10–50	Sph.	10–50 µg/mL	NA, 24 h, 37 °C	*S. aureus*, *B. subtilis*, *E. coli*	BS	[62]
37	“Green synthesis” using *Stachys lavandulifolia* extract	CuO	<80	–	80 µg/mL	PDA, 24–72 h, 28 °C	*F. solani*	FS	[63]
38	“Green synthesis” using *Syzygium alternifolium* extract	CuO	5–13	Sph.	5–80 µg/mL	NB, PDA, 24–48 h, 37 °C	*B. subtilis*, *E. coli*, *S. aureus*, *K. pneumonia*, *P. vulgaris*, *P. aeruginosa*, *S. typhimurium*, *A. solani*, *A. flavus*, *A. niger*, *P. chrysogenum*, *T. harzianum*	BS, FS	[64]
39	“Green synthesis” using *Tribulus terrestris* extract	CuO	5–22	Sph.	2.5–50 µg/mL	MHA, MHB, 24 h, 37 °C	*E. coli*, *P. aeruginosa*, *S. aureus*, *B. cereus*	BS, BC	[65]
40	Co-precipitation	CuO	108 ± 14	Sph.	1–10 µg/mL	MHA, MHB, during night, 30 °C	*P. aeruginosa*	BS, BC	[66]
41	“Green synthesis” using *Halomonas elongata* extract	CuO	57–79	Rectang.	0.28, 0.39, 0.56 mM	NA, 48 h, 37 °C	*E. coli*, *S. aureus*	BS	[67]
42	“Green Synthesis” using *Cedrus deodara* extract	CuO	–	Sph.	25–150 µg/mL	1 day, 37 °C	*S. aureus*, *E. coli*	BS	[68]
43	“Green fusion” using *Azardirachta indica* extract	Ag and Mo-dopped CuO-NPs	25, 19.8, 17.20, 14.57, 11.23	–	–	NA, 24 h, 37 °C	*P. aeruginosa*, *S. aureus*, *S. marcescens*, *C. albicans*, *A. niger*	BS, FS	[69]
44	“Green synthesis” using *Aerva javanica* extract	CuO	15–23	Sph.	50–200 µg/mL	MHA, 24 h, 37 °C; MHA, 48–72 h, 27 °C	*P. aeruginosa*, *E. coli*, *S. aureus*, *A. baumannii*, *C. albicans*, *C. krusei*, *C. tropicalis*	BS, FS, BC, FC	[70]
45	“Green synthesis” using *Mussaenda frondosa* extract	CuO	2–10	Sph.	96–593 µg/mL (MIC)	NA, 24 h, 37 °C	*S. aureus*, *B. subtilis*, *E. coli*, *P. aeruginosa*, *P. vulgaris*	BS	[71]
46	“Green synthesis” using *Ocimum tenuiflorum* extract	CuO	6–18; 4–8 × 12–44	Sph., Rod	10–50 mg/mL	NA	*B. subtilis*, *S. aureus*, *E. coli*	BS, BC	[72]
47	“Green Synthesis” using *Aloe vera* extract	CuO	5–20	Sph., Rod, Capsule	10–50 mg/mL	NA	*B. subtilis*, *S. aureus*, *E. coli*	BS, BC	[73]
48	“Green synthesis” using *Asparagus racemosus* extract	CuO	50–100 × 400–500	Rod	50 µg/mL	MHA, 24 h, 37 °C	*E. coli*, *B. subtilus*, *K. pneumonia*, *A. hydrophila*, *P. fluorescens*, *Y. ruckeri*, *F. branchiophilum*, *E. tarda*	BS	[74]
49	“Green synthesis” using *Salacia reticulate* extract/Chemical precipitation	CuO	42.2/84	–	20–80 µg/mL	MHA, 37 °C	*E. coli*, *S. aureus*, *Enterobacter*, *B. subtilis*, *P. aeruginosa*	BS	[75]
50	“Green synthesis” using *Plectranthus amboinicus* extract	CuO	5–30	Sph.	30–150 µg/mL	NA, 24 h, 37 °C	*E. coli*, *S. aureus*, *B. subtilis*, *S. pyogenes*, *P. aeruginosa*, *K. pheumoniae*, *C. albicans*, *C. tropicalis*, *A. niger*, *A. flavus*	BS, FS	[76]
51	“Green synthesis” using *Caesalpinia bonducella* extract	CuO	13.07	“Rice grain”	30/well	NA, 24 h, 37 °C	*S. aureus*, *Aeromonas* sp.	BS	[77]
52	“Green synthesis” using *Polyalthia longifolia* extract	CuO	50–60	Quasi-sph.	12.5–125 µg/mL	–	*P. aeruginosa*, *S. aureus*, *E. coli*, *S. pyogenes*; *A. niger*, *E. floccosum*, *A. clavatus*, *C. albicans*	BS, FS	[78]
53	“Green synthesis” using *Penicillium chrysogenum*	CuO	4–15, 11–53.8	Rod, Sph.	25–100 µg/mL/1.56–50 µg/mL (MIC)	LB broth, during night, 37 °C	*K. oxytoca*, *E. coli*, *S. aureus*, *B. cereus*	BS, BC	[79]
54	“Green synthesis” using *Madhuca longifolia* extract	CuO	30, 120	Unreg., Sph.	20–30 mg/mL	MHA, 24 h, 37 °C	*E. coli*, *S. aureus*, *B. subtilis*	BS	[80]
55	“Green synthesis” using *Silybum marianum* extract	CuO	15	Sph.	4, 20 mg/mL	NA, 24 h, 37 °C	*E. aerogenes*, *S. typhi*	BS	[81]
56	“Green synthesis” using *Mentha pulegium* extract	CuO	26.92 ± 4.7	Sph.	0.625–10 mg/mL	MHA, 24 h, 37 °C	*E. coli*, *S. aureus*	BS, BC	[82]
57	“Green synthesis” using *Tinospora cordifolia* extract	CuO	6–8	Spongy	500, 1000 µg/disk	NA, 48 h, 37 °C	*K. aerogenes*, *P. aeruginosa*, *E. coli*, *S. aureus*	BS	[83]
58	“Green synthesis” using *Gloriosa superba* extract	CuO	5–10	Sph.	500, 1000 µg/disk	NA, 24–36 h, 37 °C	*K. aerogenes*, *P. desmolyticum*, *E. coli*, *S. aureus*	BS	[84]
59	Commercially available NPs (Sigma-Aldrich)	CuO	48 ± 7	–	0.049–100 µg/mL	LB broth, 24 h, 37 °C/MHB, 48 h, 42 °C	*Salmonella* sp., *Campylobacter* sp.	BS, BC	[85]
60	“Green synthesis” using *Ailanthus altissima* extract	CuO	20	Sph.	20–120 µg/mL	-	*S. aureus*, *E. coli*	BS	[86]
61	Synthesis using plasma	CuO	25–160	Unreg., Sph.	25–100 µg/mL	NA, 24 h, 37 °C	*S. aureus*, *P. aeruginosa*	BS, BC	[87]
62	“Green synthesis” using *Bauhinia tomentosa* extract	CuO	22–40	Sph.	1 mg/mL	NA, 24 h, 35 °C	*E. coli*, *P. aeruginosa*	BS	[88]
63	“Green synthesis” using mint leaf extract	CuO	22–25	Cube	250 µg/mL	–	*E. coli*, *B. subtilis*	BS	[89]
64	“Green synthesis” using the endophytic fungus *Aspergillus terreus*	CuO	<100	–	1 mg/mL	MHA, 24 h, 37 °C/48 h, 28 °C	*S. typhi*, *S. aureus*, *P. mirabilis*, *P. aeruginosa*, *K. pneuemoniae*, *E. coli*, *V. cholerae*, *S. epidermidis*, *C. albicans*, *A. niger*	BS	[90]
65	“Green synthesis” using *Cassia fistula* and *Melia azedarach* extracts	CuO	43.8/28.2	Sph./Hemisph.	0.06–2 µg/mL	Blood agar, TSB 72 h, 37 °C	*K. pneumonia*, *H. pylori*	BS, AB	[91]
66	“Green synthesis” using *Brassica oleracea*/*Solanum tuberosum*/*Pisum sativum* extracts	CuO	32.5/40.7/47.2	Unreg.	35–45 µg/mL	LB agar, 24 h, 37 °C	*P. aeruginosa*, *S. aureus*, *E. coli*, *B. subtilis*	BS	[92]
67	“Green Synthesis” using *Camellia sinensis*/*Prunus africana* extracts	CuO	6/8	Sph.	30–250 µg/mL (MIC)	MHA, 24 h, 37 °C	*E. coli*, *K. pneumonia*, *S. aureus*	BS, BC	[93]
68	“Green synthesis” using *Cissus vitiginea* extract	CuO	20	Sph.	25–75 µL/disk	LB agar, 24 h, 37 °C	*E. coli*, *Enterococcus* sp., *Proteus* sp., *Klebsiella* sp.	BS	[94]
69	Pechini method	CuO, CuO/Zr (1, 3, 5, 7%)	60, 50, 40, 30, 20	–	–	MHA, 18 h, 37 °C	*E. faecalis*, *S. mutans*, *E. coli*, *S. maltophilia*	BS	[95]
70	“Green synthesis” using *Syzygium aromaticum* extract	CuO	20	Sph.	4–16 µL/disk	NA, 24 h, 37 °C; Potato dextrose agar, 72 h, 37 °C	*Bacillus* sp., *Penicillium* sp. *Pseudomonas* spp., *E. coli*; *A. niger*, *A. flavus*, *Penicillium* spp.	BS, FS	[96]
71	“Green synthesis” using *Tabernaemontana divaricate* extract	CuO	48 ± 4	Sph.	25, 50 µg/mL	MHA, 24 h, 37 °C	*E. coli*	BS	[97]
72	“Green synthesis” using *Eupatorium odoratum*/*Acanthospermum hispidum* extracts	CuO	–	Sph.	100 µL/disk	MHA, 24 h, 37 °C	*S. aureus*, *B. cereus*, *E. coli*	BS	[98]
73	Chemical deposition	CuO	–	Sheet-like, Flower-like	1.95–62.5 μg/mL	MHA with 10% blood, 72 h, 37 °C	*H. pylori*	BS, BC	[99]
74	“Green synthesis” using *Punica granatum* peel aqueous extract	CuO/Se	92.18	Sph.	8 μg/mL (MIC)	MHA with 10% blood, 72 h, 37 °C	*H. pylori*	BS	[100]
75	One-step sonochemical synthesis	CuO	50–100	Rod	25, 100 μL/L	LB agar, 24 h, 35 °C	*S. typhimurium*, *S.aureus*	BS	[101]
76	“Green synthesis” using *Balanites aegyptiaca* extract	CuO	9.79–30.8 (10–30)	Sph.	3.125–100 μg/mL	MHB, 12 h, 37 °C	*B. substilis*, *E. faecalis*, *E. coli*, *V. cholerae*	BS	[102]
77	“Green synthesis” using *Bergenia ciliata* extract	CuO	20	Sph., Hexag.	6.25, 25 μg/mL (MIC)	NA., 24 h, 37 °C	*B. subtilis*, *S. aureus*, *E. coli*, *S. typhi*	BS	[103]
78	“Green synthesis” using *Capparis decidua* extract	CuO	5–40	Sph.	50, 100 μg/mL (MIC)	–	*B. subtilis*, *S. aureus*, *E. coli*	BS	[104]
79	Co-precipitation	CuO, CuO/Ce, CuO/Nd	25.23, 27.27, 30.93	Rod., Flake, Nano-spam	20 mg/mL	MHA, 24 h	*E. aerogenes*, *B. subtilis*	BS	[105]
80	Microwave fusion	CuO, Cu_0.96_Ag_0.02_Zn_0.02_O, Cu_0.94_Ag_0.02_Zn_0.04_O, Cu_0.92_Ag_0.02_Zn_0.06_O, Cu_0.90_Ag_0.02_Zn_0.08_O	16.53, 22.17, 22.994, 24.94, 25.047	Rod, Sph. (for 0.02 Ag)	10–50 μg/mL	MHA, 24 h, 37 °C	*S. aureus*, *B. subtilis*, *E. coli*, *P. aeruginosa*	BS, BC	[106]
81	Laser ablation	CuO/Cu_2_O	<100	Sph.	–	BHI, 18 h, 37 °C	*S. enterica* subsp. *enterica* ser. *Typhimurium*, *E. coli*, *S*. *sonnei*, *Y. enterocolitica*, *V. parahaemolyticus*, *B. cereus*, *L. monocytogenes*	BS	[107]
82	“Green synthesis” using *Piper nigrum* fruit extract	CuO	60	Sph.	50, 100 μg/mL	LB, 24 h, 37 °C	*E. coli*, *S. aureus*	BS	[108]
83	Commercially available NPs from NANOTEC S.A. (Santiago, Chile)	Cu_2_O-NPs, CuO-NPs	40–70	–	100–500 μg/mL	BHI with bacitracin (0.2 units/mL), MRS-agar, 48 h, 37 °C	*S. mutans*, *S. salivarius*, *S. sanguinis*, *L. rhamnosus*	BS	[109]
84	“Green synthesis” using *Bergenia ciliata* leaf extract	CuO	50	Differ.	50–1000 μg/mL	NA, 24 h, 37 °C	*S. aureus*, *E. coli*	BS	[110]
85	“Green synthesis” using *Piper nigrum* fruit extract	CuO	37–54	Sph.	5–100 µg/mL	NA, 24 h, 37 °C	*S. aureus*, *S. epidermidis*, *S. pyogenes*, *E. coli*, *S. marcescom*, *K. pneumonia*	BS	[111]
86	Chemical deposition	CuO	2.9 ± 0.9	Quasispher.	10 μg/mL (MIC)	PDA, 4 days, 30 °C	*C. jejuni*	BS	[112]
87	“Green synthesis” using *Aegle marmelos* leaf extract	CuO	32	Differ., Rectang.	400, 800 μg/mL (MIC)	MHA, SDA, 2–18 h, 37/28 °C	*E. coli*, *S. aureus*, *C. albicans*, *C. dubliniensis*	BS, FS	[113]
88	Microwave fusion, “Green synthesis” apple peel extract	CuO	25–40	Square	25, 50 μg/mL (MIC)	MHA	*E. coli*, *S. aureus*	BS	[114]
89	“Green synthesis”, Chemical deposition	CuO	81.23	Sph.	250, 125, 31.25 μg/mL (MIC)	MHA	*E. coli*, *S. aureus*, *C. albicans*	BS, FS	[115]
90	“Green synthesis” using *Penicillium chrysogenum*	CuO	10.5–59.7	Sph., Hexag.	9–5000; 10 mg/mL	MHB, 24 h, 37 °C; PDA, 5 days, 30 °C	*S. aureus*, *P. aeruginosa*, *B. subtilis*, *S. typhimurium*, *E. coli*, *F. solani*, *F. oxysporum*, *S. sclerotia*, *A. terreus*	BS, FS	[116]
91	Chemical sol–gel method	CuO	3	–	0.5 M, 0.75 M, 1 M, 1.5 M	MHA, 24 h, 35 ± 1 °C	*P. aeruginosa*, *Staphylococcus* sp., *E. coli*	BS	[117]
92	“Green synthesis” using *Ganoderma sessile* mushroom extract	CuO	1–15	Quasisph.	0.62–19.9 μg/mL	LB agar, MHB, 24 h, 37 °C	*S. aureus*, *E. coli*, *P. aeruginosa*	BS	[118]
93	Chemical deposition	CuO, gelatin	18 ± 6, 370 ± 131	Sph.	2.5 × 10^−3^–2.5 × 10^−8^ M/L	NA, 24 h, 30 ± 1 °C	*G. candidum*, *P. digitatum*, *M. racemosus*	FS	[119]
94	Hydrothermal fusion	CuO-NPs coated with PD, PVP, PVA, PEG	834.8, 504.4, 417.9, 87.7, 266.5	Rectang., Rod-like, Brick-like	50–500 μg/mL	LB medium, 16 h, 37 °C	*E. coli*	BS	[120]
95	“Green synthesis” using cell-free supernatant of *Bacillus siamensis* HS	CuO	2–41	Sph.	50–600 μg/mL	NA, 24 h, 37 °C	*S. aureus*, *B. subtilis*, *E. faecalis*, *C*. *albicans*, *E*. *coli*, *P*. *aeruginosa*, *K. pneumoniae*, *V*. *damsela*, *Pseudoalteromonas* spp.	BS, AB	[121]
96	“Green synthesis” using *Averrhoa carambola* leaf extract	CuO	98 ± 26	Sph.	6.25–100 μg/mL	MHA, MHB, 24 h, 37 °C	*B. megaterium*, *S. aureus*, *E. coli*, *S. typhi*, *P. aeruginosa*	BS, BC	[122]
97	Chemical deposition	CuO	100	Sph.	0.01–1 g/L	–	*E. coli*	BS, BC	[123]
98	“Green synthesis” using *Centratherum punctatum* leaf extract	CuO	20–30	Sph.	9.3 mg/mL (MIC)	MHA, MHB, PDA, PDB, 16 h, 37 °C	*S. aureus*, *B. cereus*, *K. pneumonia*, *P. aeruginosa*, *E. coli*, *A. baumannii* *S. mutans*, *E. faecium*, *C. albicans*	FS, BS, AB	[124]
99	“Green synthesis” using *Stachys lavandulifolia* extract	Cu/Cu_2_O composite	80	Sph.	–	MHA, 24 h, 37 °C	*P. aeruginosa*	BS	[125]
100	Synthesis from copper sulfate pentahydrate using *Bacillus cereus*	Cu_2_O	30 ± 2	Sph.	0–500 μg/mL, 1–2 mg/mL	TSB, 24 h, 37 °C, NA, 24 h, 37 °C	*B. subtilis*, *P. aeruginosa*	BS	[126]
101	Chemical deposition	Cu_2_O/zeolite	5–30	Worm-like	150, 500 mg/L	LB agar	*E. coli*	BC	[127]
102	Laser ablation	Cu_2_O (with an admixture of Cu, CuO)	<5	Sph.	–	BHI broth	*S. aureus*	BC	[128]
103	Chemical deposition usingNaBH_4_ and N_2_H_4_	Cu_2_O/Cu_2_O + CuO/CuO	2–20	Hemisph.	–	MHA, 24 h, 35 °C	*S. aureus*, *E. coli*, *P. aeruginosa*	BS, BC	[25]
104	Chemical deposition	Cu_2_O	25.54, 25.83	Cube	10–100 μg/mL	PDA	*F. solani*	FS	[129]
105	Chemical deposition	Cu_2_O, Cu_2_O/biochar	–	–	56, 40 μg/mL (MIC)	LB agar, 24 h, 37 °C	*E. coli*	BS	[130]
106	Solvothermal synthesis	Cu_2_O	2000–6000	Cube	2 mg/mL	LB agar, 24 h, 36 °C	*B. thuringiensis*, *P. aeruginosa*	BS	[131]
107	“Green synthesis” using *Razma* seeds	Cu_4_O_3_	27	Spongy	200–600 μg/disk	NA, 36 h, 37 °C	*S. aureus*, *E. coli*	BS	[132]
108	“Green synthesis” using *Aegle marmelos* seeds	Cu_4_O_3_	200	Sph.	–	NA	*E. coli*	BS	[133]

PVA—polyvinyl alcohol; PVP—polyvinylpyrrolidone; PEG—polyethylene glycol; PD—polydopamine; GLYMO—(3-glycidyloxypropyl)-trimethoxysilane; 4-HPBA—(4-hydroxyphenylboronic) acid; BA—boronic acid; AbGram^-^—antibodies for Gram-negative bacteria; AbGram^+^—antibodies for Gram-positive bacteria; BHI—heart–brain-extract broth; MIC—minimal inhibitory concentration; MBC—minimal bactericidal concentration; BS—bacteriostatic effect; BC—bactericidal effect; AB—anti-biofilm effect; FS—fungistatic effect; LB—lysogeny broth; MHA—Mueller–Hinton Agar; NA—Nutrient Agar; NB—Nutrient broth; PDA—Potato dextrose agar; PDB—Potato dextrose broth; Rod—rod-shaped; Sph—spherical; Unreg—unregular shape; Hexag.—hexagonal shape.

## Data Availability

The raw data supporting the conclusions of this article will be made available by the authors without undue reservation.

## Supplementary Materials

The following supporting information can be downloaded at: https://www.mdpi.com/article/10.3390/ijms252111563/s1.
