# Supplementary material for: Antibacterial Properties of Copper Oxide Nanoparticles (Review)
Source: Int J Mol Sci. 2024 Oct 28;25(21):11563. doi: 10.3390/ijms252111563 (PMC11547097; doi:10.3390/ijms252111563)
Supplement: Supplementary file 1 [file ijms-25-11563-s001.zip › ijms-3251597-supplementary.pdf]

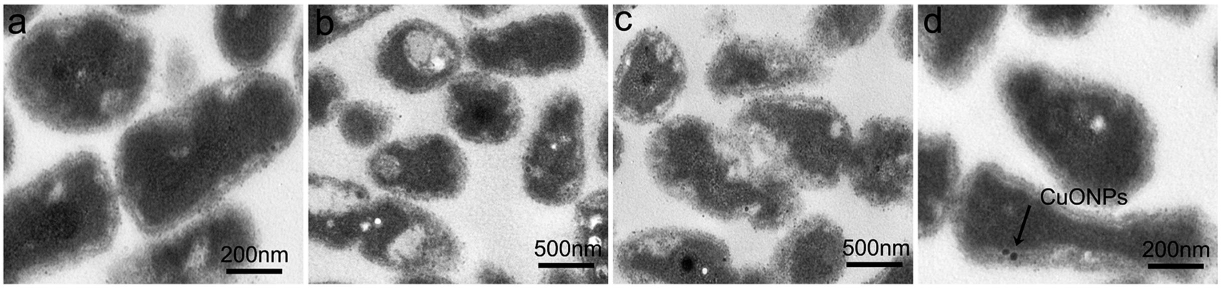

Figure S1. TEM images of *R. solanacearum* in the presence of CuONPs at (a) 0, (b) 125 μg mL^{-1}, (c) 250 μg mL^{-1} and (d) 250 μg mL^{-1} concentrations. (c) mainly represented the damaged cell structure and (d) mainly represented the absorption of CuONPs by bacterial cells (indicated by the arrows). Original image from Chen et al. [37]. The description of the image has been kept in its original form.

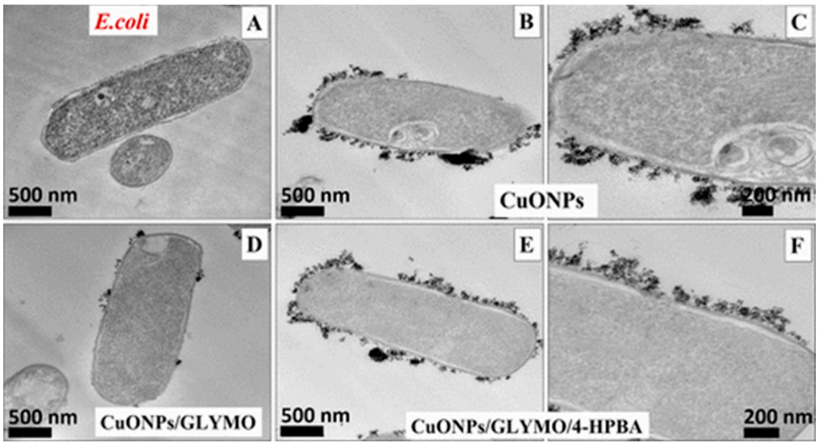

Figure S2. TEM images of *E. coli* at different magnifications: (A) before treatment and (B, C) after treatment with 25 μg mL^{-1} bare CuONPs, (D) 25 μg mL^{-1} CuONPs/GLYMO, and (E, F) 25 μg mL^{-1} CuONPs/GLYMO/4-HPBA, all for 6 h. Original image from Halbus et al. [40]. The description of the image has been kept in its original form.

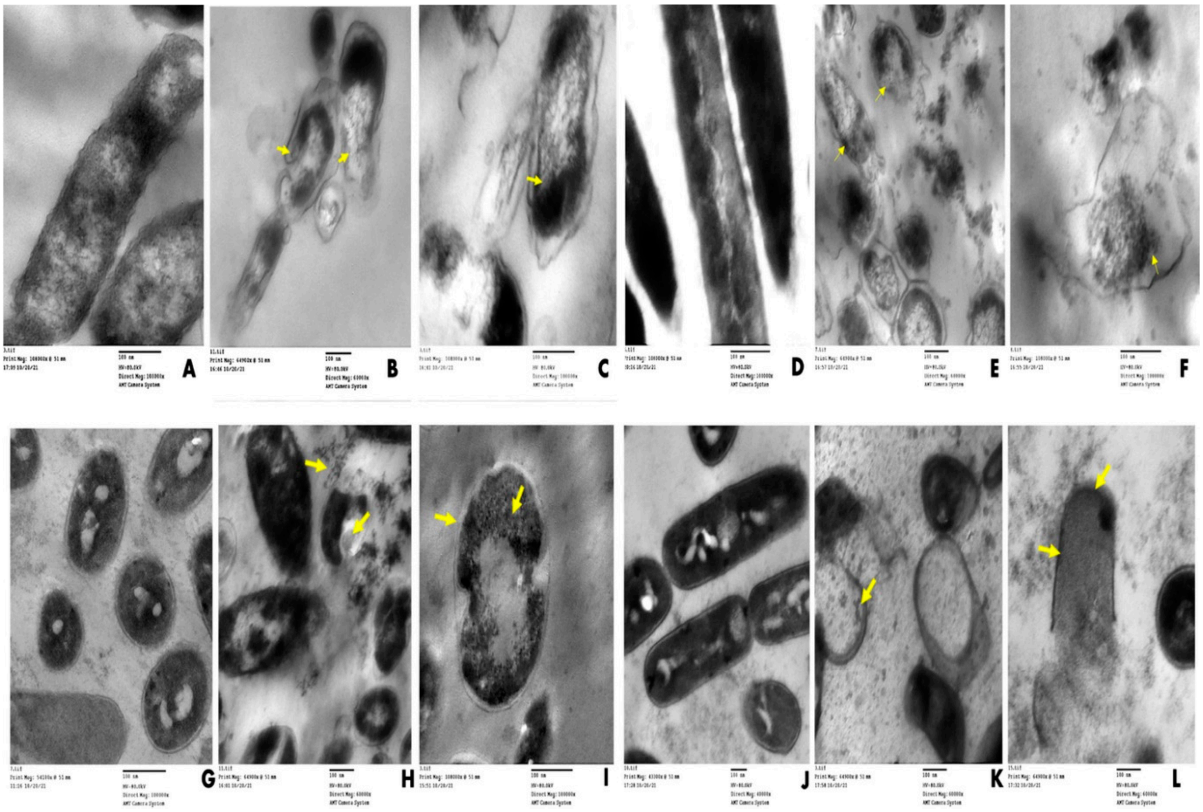

Figure S3. Transmission electron microscopy images of bacterial strains treated with CuONPs. *Escherichia coli* (**A**) control; (**B** and **C**) *Escherichia coli* affected by CuONPs (2 × MIC); *Bacillus cereus* (**D**) control; (**E** and **F**) *Bacillus cereus* affected by CuONPs (2 × MIC); *Staphylococcus aureus* (**G**) control; (**H** and **I**) *Staphylococcus aureus* affected by CuONPs (2 × MIC); *Klebsiella oxytoca* (**J**) control; *Klebsiella oxytoca* (**K** and **L**) affected by CuONPs (2 × MIC); scale bar = 100 nm. Original image from Shehabeldine et al. [79]. The description of the image has been kept in its original form.
